# Cocoa Polyphenols Modulate the Mouse Gut Microbiome in a Site-Specific Manner

**DOI:** 10.3390/nu17172876

**Published:** 2025-09-05

**Authors:** Marcus Hayden, Eliza C. Stewart, Mohammed F. Almatani, Jeremy Case, Samuel Rice, Giovanni Rompato, Korry J. Hintze, Abby Benninghoff

**Affiliations:** 1 Department of Animal, Dairy and Veterinary Sciences, Utah State University, 4815 Old Main Hill, Logan, UT 84322, USA; a02267744@usu.edu (M.H.); elizastewart@usu.edu (E.C.S.); mohammed.almatani@usu.edu (M.F.A.); jeremy.case@usu.edu (J.C.); a02273697@usu.edu (S.R.); giovanni.rompato@usu.edu (G.R.); 2 Department of Pharmacology, College of Pharmacy, King Khalid University, Al Fara, Abha 62223, Saudi Arabia; 3 Department of Nutrition, Dietetics and Food Sciences, Utah State University, 8700 Old Main Hill, Logan, UT 84322, USA; korry.hintze@usu.edu

**Keywords:** gut microbiome, cocoa polyphenol, colon mucosa, beta diversity, cecum, feces

## Abstract

**Background/Objectives:** The dietary modulation of the gut microbiome is a promising strategy for mitigating gastrointestinal diseases, such as inflammatory bowel disease (IBD) and colitis-associated colorectal cancer (CAC). Cocoa powder is rich in polyphenols, including (−)-epicatechin and (+)-catechin, which have been associated with beneficial effects on gut health and microbiome modulation. Importantly, changes in the bacterial populations associated with the gut mucosal layer may have different health impacts compared to changes in cecal or fecal microbiomes. This study investigated the effects of cocoa polyphenol supplementation on microbiome composition across the cecal, fecal, and mucosal compartments in a mouse model of colitis. **Methods:** Mice were fed either a healthy AIN93G diet (AIN) or a total Western diet (TWD), with or without 2.6% (*w*/*w*) CocoaVia™ Cardio Health Powder. Gut microbiomes from the cecum, feces, and colon mucosa were profiled using 16S rRNA sequencing at three time points: pre-, during, and post-colitis. **Results:** Microbiome composition varied substantially by site, with reduced richness and distinct taxa in the mucosal layer compared to cecal and fecal communities. The TWD significantly altered microbial composition, decreasing species evenness and shifting beta diversity. Cocoa polyphenol supplementation modulated microbial communities in a site-specific manner, increasing diversity and promoting rare taxa (e.g., Monoglobaceae, Eggerthellaceae, and RF39) primarily in cecal and fecal samples. Mucosa-associated communities were less responsive. **Conclusions:** These findings underscore the importance of the sampling site in gut microbiome research. Cocoa polyphenols exert site-selective effects, particularly in the gut lumen, highlighting the importance of considering anatomical context in dietary intervention studies aimed at improving gastrointestinal health.

## 1. Introduction

Alterations in the gut microbiome are closely linked to the development of inflammatory bowel disease (IBD) and colorectal cancer (CRC), both of which are characterized by chronic inflammation and microbial dysbiosis [1,2,3,4,5,6,7,8,9]. The composition and function of the gut microbiota influence disease progression through effects on immune signaling, epithelial barrier integrity, and microbial metabolite production [10,11,12,13]. Emerging evidence suggests that dietary interventions targeting the gut microbiome may mitigate these disease risks. Polyphenol-rich foods have been shown to modulate microbial composition, suppress inflammation, and enhance intestinal barrier function [14,15]. However, the impact of specific polyphenols, such as those found in cocoa, and their site-specific effects on distinct gut microbial niches remain poorly defined.

Western dietary patterns promote colitis and CRC through multiple mechanisms, including impaired epithelial repair, immune dysregulation, and the loss of microbial diversity [16,17]. In contrast, polyphenol-rich foods, such as blueberries, can restore microbiota diversity and reduce inflammatory damage, even in the context of high-fat diets [18]. Targeted changes to microbial communities, such as increasing anti-inflammatory taxa or reducing colitis-associated bacteria, may offer a means of restoring homeostasis and limiting disease progression [1,2,19].

Polyphenols are abundant in a range of plant-based foods, including berries, teas, cocoa, leafy greens, legumes, and nuts [20]. These compounds exhibit antioxidant, anti-inflammatory, and microbiome-modulating properties [14,15]. Cocoa, in particular, is a rich source of polyphenols, primarily (−)-epicatechin and (+)-catechin, along with theobromine and procyanidins [21,22]. Compared to other polyphenol-rich foods such as blueberries or green tea, cocoa provides a higher flavonol content per unit weight, which may translate to distinct physiological and microbial effects [21,22,23].

Cocoa supplementation has shown promise in preclinical rodent models, as cocoa-rich diets reduced the number of colonic aberrant crypt foci and suppressed pro-inflammatory signaling pathways, including IL-6 and STAT3 [24,25]. Cocoa polyphenols also enhanced colonic expression of Nrf2, a key transcription factor that regulates antioxidant enzymes and reduces oxidative stress [26]. These findings suggest that cocoa may exert protective effects against colonic inflammation and carcinogenesis. Cocoa’s influence on microbiomes has been observed across various systems. In vitro and clinical studies have reported shifts in bacterial populations, including increases in *Enterococcus* and *Lactobacillales* [27,28,29,30]. In a human trial, dietary supplementation with dark chocolate increased fecal *Lactobacillus sensu lato*, a genus associated with reduced markers of systemic inflammation [30]. However, other studies have reported a decrease in *Enterococcus* following cocoa supplementation in animal models [29], underscoring the complexity of cocoa–microbiome interactions and the influence of host and dietary context.

While these studies provide valuable insights, most have assessed the microbiome using fecal samples alone. This approach overlooks critical variation across different regions of the gut. Microbial communities differ both taxonomically and functionally between the luminal contents and the mucosal surface, as well as between sites such as the cecum and colon [31,32]. These spatial differences can influence host–microbe interactions and may impact how dietary interventions modulate inflammation or disease outcomes [33,34,35]. For example, mucosa-associated bacteria are in direct contact with host tissue and may play a more significant role in regulating immune responses than lumen-residing taxa. Understanding how dietary polyphenols affect microbiota composition at specific intestinal sites is essential for developing effective nutritional strategies.

Our prior work demonstrated that cocoa polyphenol supplementation altered gut microbiome composition, particularly in healthy mice prior to colitis induction, but did not mitigate colitis severity or inflammatory gene expression [36], highlighting a disconnect between microbiome shifts and tissue-level outcomes. In this study, we investigated the site-specific effects of dietary cocoa polyphenols on the gut microbiome in a mouse model of dextran sodium sulfate (DSS)-induced colitis. Mice were fed either a control (AIN93G) or a total Western diet (TWD), with or without cocoa polyphenol supplementation. Microbial communities were profiled from three distinct intestinal niches, including cecal contents, feces, and colon mucosa, collected before, during, and after the induction of colitis. We hypothesized that microbiome responses to both diet and cocoa supplementation would vary by site and time point, and that cocoa polyphenols would increase the relative abundance of health-associated taxa. This work aims to enhance the understanding of how cocoa polyphenols influence gut microbial communities across various intestinal sites and inform future dietary approaches for supporting gut health and managing colitis.

## 2. Methods

### 2.1. Chemicals and Reagents

Azoxymethane (AOM) was purchased from Sigma-Aldrich (St. Louis, MO, USA; CAS No. 25843-45-2). Dextran sodium sulfate (DSS; reagent grade at mol. wt. ~40 kDa) was purchased from Thermo Fisher Scientific (Waltham, MA, USA). All other chemicals were purchased at reagent grade from general laboratory supply companies.

### 2.2. Animals and Experimental Diets

The Utah State University Institutional Animal Care and Use Committee approved handling procedures for laboratory mice described herein (IACUC protocol #12680). Male C57BL/6J mice (total *n* = 240) were purchased from Jackson Laboratory at the age of 5 weeks. Mice were maintained in a specific pathogen-free vivarium at 18–23 °C, under a 12:12 h dark–light cycle, and at a constant humidity of 50%. HEPA-filtered cages were used for housing in an IVC Air Handling Solution ventilated housing system (Tecniplast, Buguggiate, Italy) with Bed-o’ Cobs^®^ ¼ bedding (Andersons, Cincinnati, OH, USA). After a week of quarantine, mice were randomized into experimental groups (*n* = 60 per group) using a random block design to standardize initial body weights across the experimental groups. Mice were housed two per cage. Cages were changed weekly, fresh food was provided twice a week, and autoclaved water was provided ad libitum throughout the study. Cages were identified by experimental group and sample time point to facilitate accurate administration of the experimental diets on the defined schedule.

Experimental diets were obtained from Envigo (Hackensack, NJ, USA). Rodent diets were purchased in two lots, mixed to account for lot variations, and stored at 4 °C for the remainder of the study. The standard AIN93G basal diet (AIN, cat. no. TD.160421) was designed by the American Institute of Nutrition to promote rodent health with an energy density of 3.8 kcal/g [37] and was used as the negative control diet. In contrast, the positive control diet was the total Western diet (TWD), designed to emulate the average American diet in terms of macro- and micronutrient density, with an energy density of 4.4 kcal/g [38]. In several previous studies, the TWD has been shown to promote gut inflammation and colon tumorigenesis [16,39,40,41].

CocoaVia™ Cardio Health Powder was obtained from Mars, Incorporated (McLean, VA, USA) and added to either AIN or TWD basal diets at 2.6% (*w*/*w*) with adjustments made to the carbohydrate and protein amounts in the basal diet to match the amount of total carbohydrates and protein as in the control diets (Table S1). Using a nutrient density scaling method, this exposure translates to approximately 1191 mg of flavanols per day in humans, which could be reasonably achieved through three daily servings of CocoaVia Cardio Health Powder.

### 2.3. Study Design

**Figure 1:**
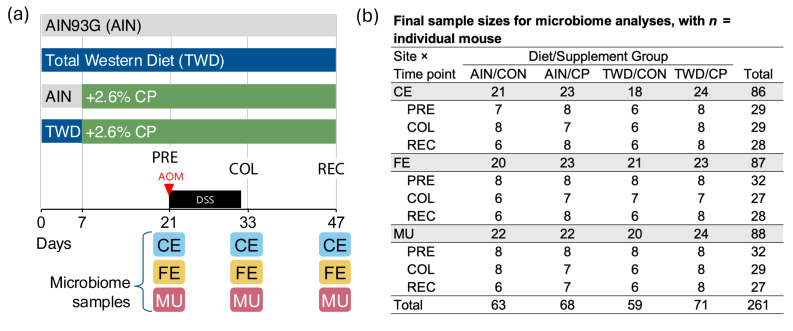
Study design and final sample sizes for microbiome analyses. (**a**) Diagram illustrates the study design, including the experimental diets and the timing of induction of colitis and collection of cecal, fecal, and mucosal samples for microbiome analysis. (**b**) The final sample sizes for each experimental subgroup following sample preparation, sequencing, and outlier detection. The individual mouse was considered the biological unit. Abbreviations include AIN, AIN93G basal diet; TWD, total Western diet; CON, control; CP, cocoa polyphenols; PRE, pre-DSS; COL, colitis; REC, recovery; AOM, azoxymethane; DSS, dextran sodium sulfate; CE, cecal; FE, fecal; MU, mucosal.

A 2 × 2 factorial design was employed for experimental factors basal diet (AIN or TWD) and supplement (CON or CP), resulting in four experimental groups: (1) AIN without cocoa supplementation (AIN/CON), (2) TWD without cocoa supplementation (TWD/CON), (3) AIN with cocoa supplementation (AIN/CP), and (4) TWD with cocoa supplementation (TWD/CP). Mice were fed the AIN or TWD basal diets for experiment days 0 to 6; then, on day 7, groups 2 and 4 were changed to AIN/CP or TWD/CP for the remainder of the study, while groups 1 and 3 continued to receive the non-supplemented diets (Figure 1). On day 22, all mice were administered AOM (10 mg/kg) intraperitoneally (i.p.) in sterile phosphate-buffered saline (PBS) and then provided with 1% (*w*/*v*) DSS in their drinking water for the following ten days. On days 21, 33, and 47, mice (*n* = 20 mice per experimental group) were randomly selected for euthanasia via CO_2_ asphyxiation. For this study, a subset of mice (*n* = 8) was necropsied to collect cecum contents, mucosa samples, and colonic fecal pellets as previously described [16]. These samples were then frozen in liquid nitrogen and stored at −20° Celsius until further analysis. The remainder of the mice were euthanized and necropsied for other analyses, as reported previously [36].

Power analysis was performed using the micropower R package (https://github.com/brendankelly/micropower, accessed on 1 September 2025), which first simulates beta diversity distance matrices given population parameters computed from prior studies and then simulates a range of effect sizes and rarefaction curves to estimate PERMANOVA power from the simulated distance matrices [42]. With *n* = 6 or 8 mice/group with variance of 0.12, we have >80% power to detect an effect size (ω^2^) of 0.018 or 0.021, respectively. These effect sizes are similar to that for other studies, as reported by Kelly et al. [42].

### 2.4. Microbiota Profiling

The complete procedure for sample processing and sequencing is found in Rodriguez et al. [41], with minor modifications. Samples were blinded to the experimental group before processing, with only the project director (A.D.B.) and student researchers (M.H. and E.C.S.) having access to the sample identification code and experimental group key. Samples remained blinded throughout the preparation, sequencing, and data processing stages.

DNA was extracted from cecal, fecal, and mucosal samples using the QIAmp DNA Stool Mini Kit (Qiagen, Frederick, MD, USA) and diluted to 5 ng/µL in TE buffer (pH 8.0). The V4 region of the 16S rRNA gene was amplified using 515F and 806R primers (Integrated DNA Technologies, Coralville, IA, USA) with the Platinum HS PCR kit (Thermo Fisher, Waltham, MA, USA), followed by a second PCR for sample barcoding. Amplicons were purified using AMPure XP beads (Beckman Coulter, Indianapolis, IN, USA), quantified with the Quant-IT PicoGreen dsDNA assay (Thermo Fisher), and pooled to a final concentration of 2 nM. Samples with insufficient starting material or that failed to amplify were excluded from further analysis. Sequencing was performed at the Utah State University Genomics Core using the Illumina MiSeq platform with the MiSeq Reagent Kit V2 (2 × 250 bp, 500 cycles; Illumina, San Diego, CA, USA).

### 2.5. Microbiome Sequencing Data Analysis

Sequencing output was processed through the QIIME2 [43] and DADA2 [44] pipelines to filter for quality and length, to remove chimeras, and to create a table of amplicon sequence variants (ASVs). The SILVA classifier (silva-138-99-515-806-nb-classifier.qza), specific to the V4 region, was used to assign taxonomy to the ASVs [45]. The Marker Data Profiling module in Microbiome Analyst hosts several analyses conducted in this study [46]. Three samples with fewer than 5000 sequence reads were excluded from the analysis to avoid an artificially low count value for rarefaction, which would have limited the capacity to assess rare taxa. Sequence count data were processed to remove samples with low counts (a minimum of four counts with a 20% prevalence) and low variance (10% based on the interquartile range). Data were then rarefied to the minimum library size and normalized by total sum scaling (File S1).

Statistical analysis of bacterial relative abundance at the phylum and family levels were performed in a stepwise manner. First, multifactor models were applied to the entire dataset using MaAsLin2, which fits a general linear model to each microbial feature, including experimental factors (time point, site, basal diet, and supplement) and controls for covariates as fixed effects in the model [47]. This analysis identified the main effects of each factor across the complete dataset. Second, pairwise analyses were conducted within data subsets by site and time point to assess the impacts of diet and supplement. False discovery rate (FDR) adjusted *p*-values are reported, and an FDR *p*-value < 0.05 was considered statistically significant. Alpha diversity was evaluated using observed ASVs and Shannon diversity indices. Alpha diversity scores were analyzed for the entire dataset to identify potential outlier microbiome profiles by applying the robust outlier test (ROUT) with a conservative *Q* value of 0.1% (GraphPad Prism v. 10.0.1, San Diego, CA, USA), meaning that there is a≤ 0.1% chance of excluding a data point as an outlier in error; this approach was determined a priori. No outlier samples were identified.

Differences in alpha diversity and Bacillota–Bacteroidota (B:B) ratios (formerly named Firmicutes–Bacteroidetes) were assessed by generalized linear models followed by Tukey HSD post hoc tests (JMP v.17.1.0, SAS Institute, Cary, NC, USA). Outliers were identified using the robust outlier test (ROUT) with a *Q* value of 0.1% (GraphPad Prism). Beta diversity was assessed using unweighted and weighted UniFrac distances with principal coordinates analysis (PCoA), and PERMANOVA to determine clustering significance with *R*^2^ > 0.10 and *p*-value < 0.05 considered significant; pairwise PERMANOVA tests among conditions for each experimental factor were also determined, with the FDR-adjusted *p*-value < 0.05 considered significant.

During the initial data analysis, we identified 66 out of 1708 ASVs that did not map to known taxa in the SILVA database and were designated as “not assigned”. These ASVs were primarily found in colon mucosa samples (Figure S1) and initially appeared to represent unannotated, mucosa-associated taxa. To validate their status, we cross-referenced these sequences against the NCBI nucleotide database, which revealed that nearly all aligned with the mouse genome. This is consistent with the expectation that mucosal samples contain host DNA and that the bacterial V4 primers may also amplify some mouse sequences. Because the study focused on the gut microbiome rather than host sequences, inclusion of these unassigned ASVs would have artificially skewed beta diversity analyses and confounded interpretation. We therefore excluded them from the dataset and repeated all analyses, which are reported herein.

### 2.6. Microbiome Functional Prediction

The functional capacity of microbiomes was predicted using tax4fun2 [48] to generate gene abundance tables based on a minimum 16S rRNA sequence similarity, which were then processed using the Microbiome Analyst Shotgun Data Profiling module, with data filtering and normalization as described in Section 2.5. The dataset was then analyzed using MaAsLin2 to identify those KEGG orthology terms that were differentially abundant for each of the experimental factors, including site, time point, basal diet, and cocoa supplement, based on an FDR-adjusted *p* < 0.05. The lists of significant terms were then subject to pathway association analysis using the globaltest algorithm to identify enriched functional pathways (FDR *p* < 0.05).

## 3. Results

### 3.1. Microbiome Sequencing

A total of 1.2 × 10^7^ amplicons were sequenced. After filtering for length, quality, and abundance and inspecting for chimeras, 7.9 × 10^6^ sequences were assigned to ASVs (Silva database version 138SSU) for an average of 30,235 sequences per sample assigned to 1640 ASVs. After filtering for low prevalence and variance, the sequence library was rarefied to a depth of ~6000 sequences (Figure S2).

### 3.2. Microbiome Taxonomic Composition

**Figure 2:**
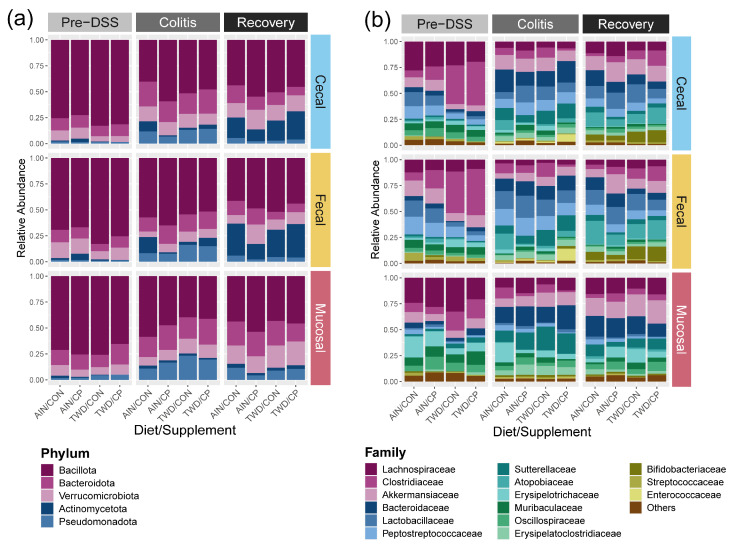
Taxonomic classification of murine cecal, fecal, and mucosal microbiomes at each time point. Data are shown as the relative normalized abundance of bacteria at the (**a**) phylum and (**b**) family (top 15 shown) taxonomic levels for each diet/supplement condition (*n* = 6 to 8 for each experimental subgroup). Abbreviations include AIN, AIN93G basal diet; TWD, total Western diet; CON, control; CP, cocoa polyphenols. For reference, prior Phyla names were Firmicutes for Bacillota, Bacteroidetes for Bacteroidota, Verrucomicrobia for Verrucomicrobiota, Actinobacteria for Actinomycetota, and Proteobacteria for Pseudomonadota.

Significant differences in bacterial phyla were primarily driven by the time point, with pronounced shifts observed during colitis and incomplete restoration by recovery (Figure 2a and Figure S3). Site- and diet-specific effects were more modest and variable, and CP supplementation had limited influence at the phylum level. A complete presentation of results for bacterial abundance at the phylum taxonomic level is provided in the Supplementary Materials (Figures S3–S6).

**Figure 3:**
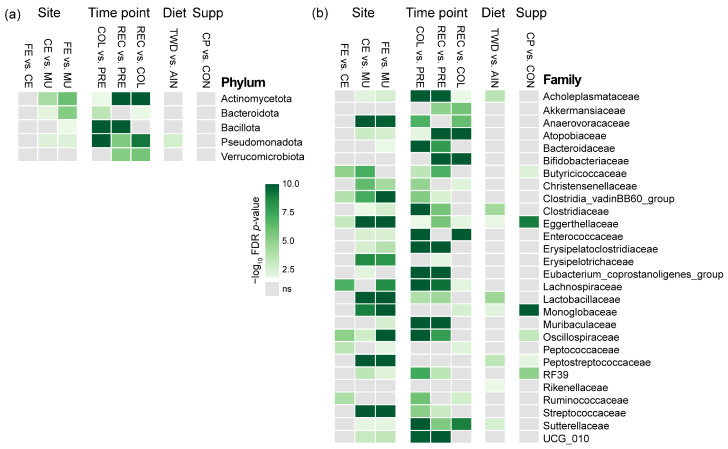
Summary of the FDR-adjusted *p*-values for MaAsLin2 multivariate analysis of bacteria relative abundance for (**a**) phyla and (**b**) families. The heatmap shows the −log_10_ FDR *p*-values for the main effects of experimental site (*n* = 86 to 88), time point (*n* = 83 to 93), basal diet (*n* = 130 to 131), and cocoa polyphenol supplement (*n* = 122 to 139). A subsequent analysis included pairwise tests for diet and supplement within each experimental site and time point, as reported in File S2. Abbreviations include CE, cecal; FE, fecal; MU, mucosal; PRE, pre-DSS; COL, colitis; REC, recovery; AIN, AIN93G basal diet; TWD, total Western diet; CON, control; CP, cocoa polyphenols.

**Figure 4:**
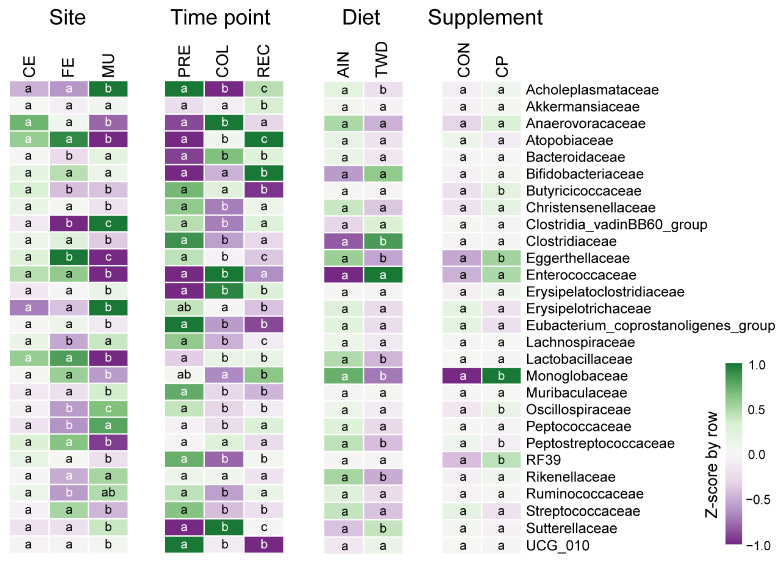
Comparisons of microbiome relative abundance for main effects experimental factors. Heatmaps show the Z-scaled (by row) average relative abundance of bacteria in the indicated family taxonomic groups for site (*n* = 86 to 88), time point (*n* = 83 to 93), basal diet (*n* = 130 to 131), and cocoa polyphenol supplement (*n* = 122 to 139). Different letters indicate that the compared groups are significantly different (FDR *p* < 0.05) (within rows) for each factor (while controlling for other factors) as determined by MaAsLan2 multivariate analysis. Complete results are available in File S2. Abbreviations include CE, cecal; FE, fecal; MU, mucosal; PRE, pre-DSS; COL, colitis; REC, recovery; AIN, AIN93G basal diet; TWD, total Western diet; CON, control; CP, cocoa polyphenols.

Microbiome composition at the family level differed significantly by site (Figure 3, Figure 4, Figures S7 and S8; File S2). The mucosal microbiota was enriched for Erysipelotrichaceae (9.8% vs. 1.8% cecal, 2.3% fecal; *p* < 10^−8^), Clostridia_vadinBB60_group (1.7% vs. 0.43% cecal, 0.15% fecal; *p* < 10^−7^), Oscillospiraceae (6.7% vs. 2.8%; *p* < 0.01), and Sutterellaceae (9.5% vs. 5.6%; *p* < 0.05) (Figure S7a). More modest increases were observed for Lachnospiraceae and Bacteroidaceae, though only in mucosa vs. feces. In contrast, lumen-associated taxa such as Lactobacillaceae (1.7% mucosal vs. 10.5% cecal, 13.2% fecal; *p* < 10^−12^), Peptostreptococcaceae (*p* < 10^−24^), and Atopobiaceae (*p* < 0.01) were depleted in the mucosa. Cecal and fecal profiles were broadly similar, with some differences in Butyricicoccaceae, Lachnospiraceae, and Oscillospiraceae. These patterns indicate a distinct mucosal community enriched for intestinal wall-associated lineages.

Across timepoints, disease progression drove marked shifts in the abundance of several families (Figure 3, Figure 4 and Figure S7b). Enterococcaceae increased sharply during colitis (from 0.003% to 2.5%, *p* = 1.2 × 10^−41^) and declined at recovery (0.04%). Anaerovoracaceae and Erysipelatoclostridiaceae also rose during colitis and remained elevated at recovery, while Muribaculaceae declined significantly (*p* = 4.5 × 10^−14^) and did not recover. Sutterellaceae peaked during colitis (2.1% to 14%, *p* = 2.0 × 10^−24^) and declined at recovery (5.4%), a level distinct from both pre-DSS and colitis (*p* < 10^−6^). In contrast, Akkermansiaceae and Bifidobacteriaceae reached their highest abundance during recovery, increasing significantly from colitis levels (*p* < 10^−6^ and *p* < 10^−27^, respectively).

Bacterial community shifts in response to colitis and recovery were highly site-specific (Figure S8; File S2). In the cecal and fecal compartments, inflammation drove sharp increases in Sutterellaceae and Enterococcaceae (e.g., cecal Sutterellaceae, *p* = 3.35 × 10^−9^; fecal Enterococcaceae, *p* = 3.67 × 10^−16^), alongside declines in Lachnospiraceae and Muribaculaceae, which rebounded post-recovery. The mucosal microbiome was more stable, with limited shifts across time points. Notable exceptions included increases in Akkermansiaceae and Atopobiaceae during recovery (*p* = 3.22 × 10^−4^ and *p* = 2.07 × 10^−5^, respectively). These findings highlight greater microbial plasticity in luminal communities compared to the mucosal niche.

**Figure 5:**
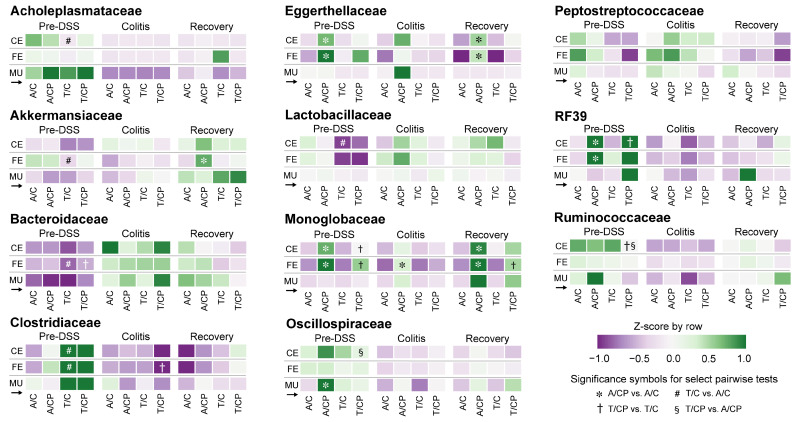
Impact of diet and supplement on microbiome relative abundance. Heatmaps show the Z-scaled (by row) relative abundance of selected bacteria families for each sample site and study time point. Four pairwise comparisons were selected a priori, with significant differences (FDR *p* < 0.05) as indicated in the figure legend. Significance was determined by MaAsLin2 multivariate analyses (*n* = 6 to 8 for each diet × supplement subgroup). Complete results are provided in File S2. The small black arrow indicates that the heatmaps should be read across rows. Abbreviations include CE, cecal; FE, fecal; MU, mucosal; PRE, pre-DSS; COL, colitis; REC, recovery; A, AIN93G basal diet; T, total Western diet; C, control; CP, cocoa polyphenols.

The basal diet had a notable impact on the microbiome, particularly before DSS treatment (Figure 3, Figure 5, Figures S10 and S11). In both cecal and fecal samples, the TWD reduced Peptostreptococcaceae and Akkermansiaceae, and increased Clostridiaceae (*p* < 0.05). In the cecum, TWD also reduced Lactobacillaceae (from 12% to 2.3%, *p* = 0.05) and Acholeplasmataceae (from 0.66% to 0.21%, *p* = 0.01) prior to the onset of colitis.

CP supplementation influenced specific low-abundance families, particularly in the cecum and feces (Figure 3, Figure 5, Figures S10 and S11). Monoglobaceae abundance increased consistently with CP across all timepoints (*p* < 0.05), and RF39 (order Bacilli) was elevated with CP at Pre-DSS (*p* < 0.05). Eggerthellaceae also increased with CP in both sites at Pre-DSS and Recovery (*p* < 0.05). In the mucosa, CP-associated effects were limited, with a single significant increase in Oscillospiraceae in AIN-fed mice before DSS (5.2% in control vs. 12% with CP, *p* = 0.031). Overall, CP-related changes were most evident in luminal compartments and at the pre-DSS and recovery phases.

### 3.3. Firmicutes–Bacteroidota Ratio

The log_10_ F:B ratio varied significantly by time point (*p* < 0.0001) and by site (*p* < 0.0001), irrespective of diet or supplement (Figure S12a; Tables S2 and S3). At pre-DSS, the ratio was highest (0.82 ± 0.25), significantly greater than at colitis (0.46 ± 0.47, *p* < 0.0001) and recovery (0.55 ± 0.41, *p* < 0.0001). The mucosal site had a lower F:B ratio (0.48 ± 10.43) compared to the cecal (0.63 ± 0.38, *p* = 0.0100) and fecal sites (0.74 ± 0.38, *p* < 0.0001). No significant differences were found for diet and supplement variables alone (*p* > 0.05) or when compared for diet × supplement within each time point and site (Figure S12c).

### 3.4. Microbiome Alpha Diversity

Alpha diversity was assessed using observed ASVs and the Chao1 index for species richness, as well as the Shannon diversity index for species evenness. Results for Chao1, consistent with observed ASVs, are provided in Figure S13 and Tables S4–S6. First, we considered the differences in alpha diversity across different sites. Observed ASVs were notably higher in cecal samples (79.4 ± 20.8) than in fecal (69.1 ± 22.2) or mucosal (52.73 ± 20.8) samples, all significantly different from each other (*p* < 0.0001) (Figure S13a). However, the Shannon index revealed greater diversity in the cecal (2.82 ± 0.44) and mucosal (2.70 ± 0.57) microbiomes compared to fecal (2.55 ± 0.36) samples (*p* < 0.05), with no significant difference between cecal and mucosal sites. Next, both observed ASVs and the Shannon index indicated that gut microbiome diversity varied significantly with time point, showing the greatest reduction during active colitis compared to pre-DSS (*p* < 0.001) and partial recovery post-injury (*p* < 0.001) (Figure S13). Additionally, we observed significant effects of diet and supplementation. CP supplementation increased species richness compared to CON-fed mice (*p* = 0.0006). Mice fed the AIN basal diet had higher species evenness than those provided the TWD (*p* = 0.0129).

**Figure 6:**
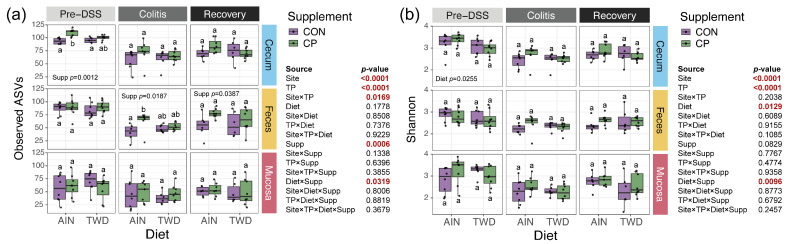
Alpha diversity measures for mouse cecal, fecal, and mucosal microbiomes at each experimental time point are shown as (**a**) observed ASVs and (**b**) Shannon index scores. Data are shown as box-and-whisker plots showing the median ± the interquartile range (*n* = 6 to 8 for each diet × supplement subgroup). Different letters indicate that the experimental diet groups are statistically different (*p* < 0.05, indicated by bold red text), as determined by a generalized linear model with a Tukey HSD post hoc test, as described in Section 2. Within some time point × site plots, the *p*-values for significant main effects of diet or supplement are provided. Main effects and interactions for site, time point, diet, supplement, and all interactions are provided as inset tables for both measures of alpha diversity. Complete statistical results, including the Chao1 index, are provided in Tables S4–S6.

Next, we examined the effects of diet and supplementation within each time point and microbiome site (Figure 6a). The highest species richness was observed in the cecal microbiome of mice on the AIN/CP diet at the pre-DSS time point (110 ± 6.9), significantly higher than their AIN/CON counterparts (90.1 ± 13.6, *p* = 0.0092). Despite an overall reduction in bacterial numbers during colitis, AIN/CP-fed mice showed significantly higher richness (63.1 ± 18.5) compared to AIN/CON (41.5 ± 15.3) in fecal samples during active colitis (*p* = 0.0306). This significant main effect of supplementation (*p* = 0.0387) persisted in fecal microbiomes through recovery. However, no effect of CP was noted for mucosal bacterial communities at any time point, nor were any significant effects of CP observed at any time point or site for the Shannon index (Figure 6b).

### 3.5. Microbiome Beta Diversity

The beta diversity of the samples was assessed using unweighted and weighted UniFrac metrics to compare the overall microbiome composition among samples. Considering the complete dataset, the main experimental factors leading to notably distinct microbiomes were time point and microbiome site (Figure S14), with similar cluster patterns evident for both unweighted and weighted analyses (*p* = 0.001). Microbiome communities in the cecum and feces were broadly similar, particularly during active colitis and recovery (*p* > 0.05 for all PERMANOVA pairwise tests). However, these luminal microbiome populations were quite distinct from the mucosal microbiome (Figure S10) (*p* ≤ 0.002 for all PERMANOVA pairwise comparisons). Considering the effect of time point, bacterial profiles differed most significantly prior to DSS treatment, with greater overlap evident for communities at the colitis and recovery time points. Alternatively, diet- or supplement-driven changes in the microbiome were not sufficiently robust to be apparent when considering the entire dataset, irrespective of sample site or time point, for either unweighted or weighted UniFrac analyses (*R^2^* < 0.1) (Figure S14).

Principal coordinates analysis of UniFrac distances showed that microbial communities varied significantly by time point within each site (Figure S15a). The strongest separation by time was observed in the cecal compartment (unweighted *R*^2^ = 0.28, *p* < 0.001), while fecal and mucosal communities exhibited weaker but significant temporal clustering, especially at recovery (mucosal weighted *R*^2^ = 0.18, *p* = 0.005). Conversely, site-based differences were most pronounced at the pre-DSS and recovery time points (Figure S15b). At both stages, mucosal communities clustered distinctly from fecal and cecal samples (e.g., pre-DSS weighted *R*^2^ = 0.22, *p* < 0.001), but this site-based structure diminished during colitis, indicating convergence across compartments during inflammation. These patterns underscore both the compartment-specific and temporally dynamic nature of microbiome composition in response to colitis.

**Figure 7:**
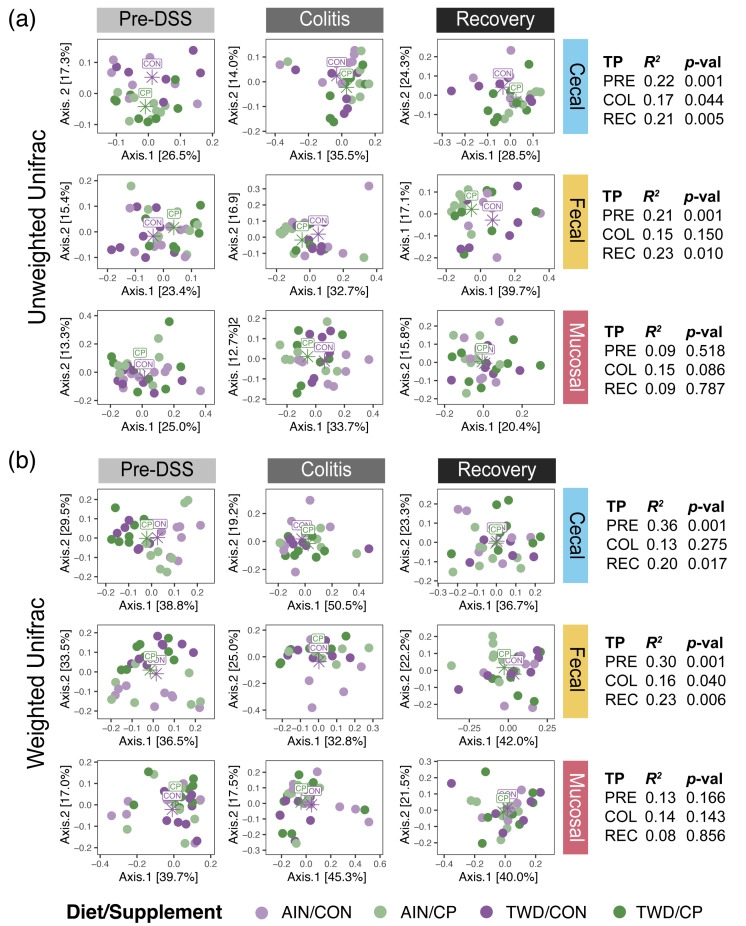
Beta diversity of mouse cecal, fecal, and mucosa microbiomes at each time point is shown as (**a**) unweighted UniFrac and (**b**) weighted UniFrac distances. Data are shown as principal coordinate analysis plots using the first two coordinates, with centroids for CON and CP supplement groups shown (*n* = 6 to 8 for each diet × supplement subgroup). The overall PERMANOVA R^2^ and *p*-values (*p*-val) for each site and time point are provided to the right, and PERMANOVA results for TWD vs. AIN and CP vs. CON within each site and time point are provided in Table S7. Abbreviations are TP, time point; Pre, Pre-DSS; COL, Colitis; and REC, Recovery; AIN, AIN93G diet; TWD, total Western diet; CON, control; and CP, cocoa polyphenols.

Therefore, we next examined the impact of the basal diet and CP supplement on microbial diversity at each time point for each sample site (Figure 7). At the pre-DSS time point, unweighted UniFrac analysis indicated a clear organization of samples according to the supplement for both the cecal and fecal microbiomes (Figure 7a, Table S7) (*R*^2^ > 0.1, *p* = 0.001). Alternatively, weighted UniFrac analysis revealed much stronger clustering by the basal diet, as illustrated by the difference in symbol shading (Figure 7b) (*R*^2^ > 0.2, *p* = 0.001). This differential response suggests that cocoa supplementation may support rare species, whereas diet has a broader effect on the luminal microbiome. These apparent clusters, driven by supplement for unweighted and diet for weighted UniFrac beta diversity, were much less apparent during active colitis but were somewhat restored by the recovery time point for the unweighted analysis (*R*^2^ > 0.1, *p* < 0.010 for cecal and fecal microbiomes) (Figure 7a). In contrast, the beta diversity of the mucosal microbiome was not significantly affected by either basal diet or CP supplementation at any time point (*R*^2^ < 0.1, *p* > 0.05).

### 3.6. Functional Prediction

**Figure 8:**
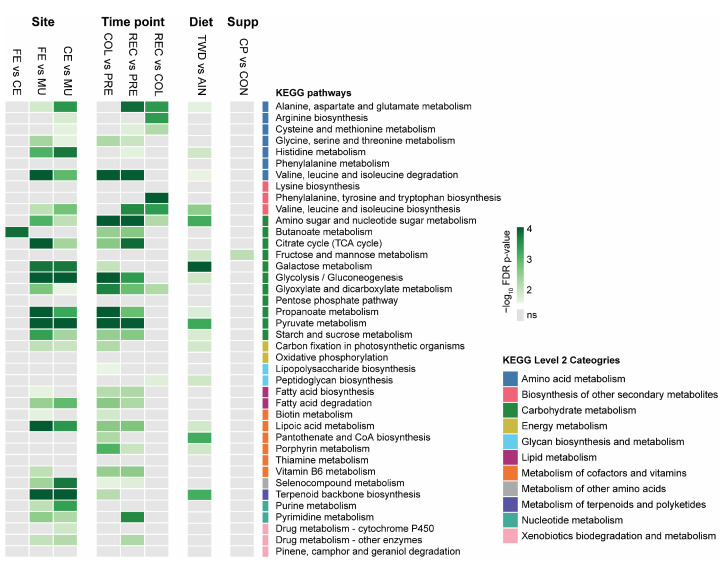
Predicted functional capacity of gut microbiomes. The heatmap depicts the -log_10_ FDR *p*-values for main effects of site (*n* = 86 to 88), time point (*n* = 83 to 93), basal diet (*n* = 130 to 131), and cocoa polyphenol supplement (*n* = 122 to 139) for all KEGG level 3 pathways identified as significantly enriched (FDR *p* < 0.05) for any experimental condition. Cells colored gray were not significant or not reported in the enriched pathway analysis results. Higher-level 2 KEGG category annotations are also provided. Enriched pathways were identified following MaAsLin2 multivariate analysis of KEGG term counts mapped to each sample using tax4fun2 in Microbiome Analyst. Gray cells indicate that the pathway was not in the statistical results report. Complete results of the pathway enrichment analysis are available in File S3. Abbreviations include CE, cecal; FE, fecal; MU, mucosal; PRE, pre-DSS; COL, colitis; REC, recovery; AIN, AIN93G basal diet; TWD, total Western diet; CON, control; CP, cocoa polyphenol.

Tax4fun2-based functional predictions revealed distinct metabolic profiles across fecal, cecal, and mucosal microbiomes. In the fecal vs. mucosal comparison, 25 pathways differed significantly (*p* < 0.05), with elevated levels of glycolysis/gluconeogenesis, pyruvate metabolism, propanoate metabolism, and amino acid degradation pathways in fecal samples (Figure 8 and Figure S16; File S3). The comparison between cecal and mucosal samples showed 23 significantly different pathways (*p* < 0.05), including higher glycolysis/gluconeogenesis, pyruvate metabolism, and histidine metabolism in cecal samples. In contrast, the fecal and cecal communities were functionally similar, with only butanoate metabolism showing a significant difference (*p* = 1.6 × 10^−4^). These results highlight substantial functional differences between mucosal and luminal microbiomes, with minimal divergence between proximal and distal luminal sites.

Tax4fun2 analysis identified significant shifts in predicted microbial function across disease time points. Comparison of colitis to pre-DSS samples revealed 23 significantly different pathways (*p* < 0.05) (Figure 8 and Figure S17), including increased glycolysis/gluconeogenesis, pyruvate and propionate metabolism, amino sugar metabolism, and valine, leucine, and isoleucine degradation during active inflammation. The recovery vs. colitis comparison showed nine significant differences, many of which overlapped with colitis-associated pathways but at reduced enrichment, indicating a partial reversal of inflammation-related signatures. Recovery vs. pre-DSS comparisons identified 24 differentially abundant pathways, reflecting ongoing divergence from baseline despite clinical recovery. Together, these findings demonstrate substantial functional remodeling of the microbiome during colitis, followed by incomplete resolution during recovery.

Functional pathway analysis revealed that basal diet exerted a stronger influence on predicted microbial function than CP supplementation (Figure 8 and Figure S18a). Comparison of the TWD versus the AIN basal diet identified 16 significantly different pathways (*p* < 0.05), reflecting broad functional remodeling. In contrast, only one pathway, fructose and mannose metabolism, differed significantly between CP and CON groups (*p* = 0.0132) (Figure S18b). These findings suggest that while the basal diet composition substantially alters microbial functional potential, CP supplementation has a more limited impact under the tested conditions.

## 4. Discussion

This study investigated the effect of cocoa polyphenol supplementation on the gut microbiome across distinct sites of the murine lower gastrointestinal tract in an AOM/DSS-induced colitis model fed a Western-type diet. While polyphenol-rich foods have been shown to improve gut barrier function [49], reduce colitis severity [50], and modulate microbial composition [21], few studies have examined how these effects vary by gut location during inflammatory disease. Our findings demonstrate that cocoa supplementation is associated with increased species richness and distinct taxonomic shifts in the cecal and fecal microbiomes, but not in the mucosa. These results underscore the limitations of using fecal samples alone to assess dietary impacts and highlight the need to consider both luminal and mucosal communities, particularly given the mucosa’s close interaction with the colonic epithelium and relevance to colorectal cancer etiology [35].

Building on previous findings in inflammatory bowel disease models [35,51], this study compared microbial communities across three disease stages: pre-colitis, active colitis, and recovery. The study time point had a strong effect on alpha diversity, with colitis inducing the lowest richness (observed ASVs) and evenness (Shannon index), patterns commonly seen in Crohn’s disease and ulcerative colitis [52,53]. Although diversity increased during recovery, it did not return to the baseline level, suggesting a lasting disruption that extends beyond visible symptoms. Colitis also reduced the ability to distinguish microbiomes by diet or supplement, particularly at the cecal and fecal sites, where group differences re-emerged post-recovery. These results highlight the microbiome’s dynamic response to inflammation and emphasize the need to account for disease stage in dietary intervention studies.

Consistent with these observations, the companion publication by Stewart et al. [36] reported parallel outcomes for disease activity, histopathology, and mucosal gene expression in the same experimental animals. That study demonstrated that cocoa polyphenol supplementation did not alleviate colitis severity, as disease activity index scores and histological measures of mucosal inflammation and injury remained elevated, particularly in mice fed the Western-type diet. Likewise, transcriptional profiling of over 700 immune- and cancer-related genes revealed a robust inflammatory signature during colitis and recovery, including induction of cytokines (e.g., *Tnf*, *Cxcl1*, *Ccl2*), innate immune receptors (e.g., *Tlr2*, *Il1r1*), and barrier-associated transcripts (e.g., *Icam* and *Vcam*), but these responses were not suppressed by cocoa supplementation. Instead, basal diet emerged as the dominant factor, with Western diet animals exhibiting a more pro-inflammatory baseline gene expression profile prior to colitis. Together, these findings emphasize that the microbiome shifts reported here, particularly the enrichment of rare taxa in cecal and fecal communities, occurred in the absence of measurable improvements in mucosal injury or immune activation, highlighting the complex and context-dependent relationship between dietary polyphenols, the gut microbiome, and host physiology.

Microbial diversity varied significantly across gut sites. The cecal microbiome exhibited the highest species richness, while the mucosal site was the least diverse but showed greater evenness than the fecal samples. Site-specific variation was also shaped by time, with significant interactions between site and disease stage. Notably, the mucosal microbiome showed no significant response to diet or cocoa supplementation, suggesting either that the administered cocoa dose was insufficient to affect this niche or that its impact does not manifest in diversity metrics. In contrast, the cecal and fecal microbiomes showed distinct clustering by diet and supplement, particularly pre-colitis. Cocoa appeared to enrich rare taxa, while basal diet drove broader community shifts, highlighting the site-specific nature of dietary modulation.

These spatial patterns are not only consistent with prior reports of gut biogeography [31,32,54], but also align with the broader conceptual framework proposed by Tropini et al. [55], which emphasizes how physical and biochemical gradients along the gastrointestinal tract shape microbial community structure. Our observation that the cecal and fecal microbiomes were taxonomically and functionally similar, while mucosal communities were distinct, supports the idea that luminal environments foster higher microbial diversity due to relatively stable nutrient availability and limited immune exposure. Conversely, the colon mucosa represents a more selective and competitive niche, influenced by oxygen diffusion, host antimicrobial factors, and direct epithelial contact. These conditions likely contribute to the reduced richness, enrichment for mucus-adapted taxa, and limited response to dietary supplementation observed in our mucosal samples.

Tropini and colleagues further noted that inflammation, dietary shifts, and altered gut motility can disrupt this spatial structuring, leading to altered microbial partitioning across gut niches [55]. Consistent with this model, we found that colitis significantly reduced alpha diversity across all sites and temporarily diminished the site- and diet-specific distinctions evident under baseline conditions. Beta diversity and functional predictions suggest that spatial organization partially re-emerged during recovery, particularly in the cecal and fecal microbiomes, which showed greater resilience. In contrast, the mucosal community remained less responsive and more taxonomically constrained, consistent with its close integration with host defenses and slower ecological turnover.

Functional prediction analysis further reinforced this spatial differentiation, revealing distinct metabolic capacities across gut sites and conditions. Luminal communities (feces and cecum) were enriched in carbohydrate fermentation and short-chain fatty acid pathways, including butanoate, propanoate, and pyruvate metabolism, which are well-suited to the fiber- and starch-rich environment of the intestinal lumen [54]. In contrast, mucosal communities exhibited reduced fermentative capacity and showed enrichment in stress response and cell wall biosynthesis functions, reflecting adaptation to immune-exposed and oxygen-permeable interfaces [56,57]. Minimal functional divergence was observed between fecal and cecal communities, with butanoate metabolism being the only significantly different pathway, supporting the use of fecal samples as a reasonable proxy for luminal function in murine models.

Inflammation emerged as a major driver of metabolic disruption. During active colitis, microbial functions shifted toward glycolysis, amino sugar metabolism, branched-chain amino acid degradation, and the activity of ABC transporters. These functions are typically associated with facultative anaerobes, such as Enterobacteriaceae, that exploit host-derived sugars and nitrate in inflamed environments [58,59]. While these signals declined during recovery, they remained elevated compared to pre-colitis levels, indicating persistent metabolic disturbance [60].

The basal diet had a pronounced effect on microbial function. Mice fed the TWD exhibited increased enrichment in sugar metabolism pathways, including galactose, fructose/mannose, and amino sugar metabolism, as well as pathways tied to pyruvate metabolism, pantothenate and CoA biosynthesis, and terpenoid backbone synthesis. These shifts reflect adaptation to high-fat, high-sugar diets and align with reports linking Western dietary patterns to reduced fiber fermentation and increased mucin degradation [60,61,62,63]. In contrast, cocoa supplementation produced a more focused metabolic response. The only significantly altered pathway was fructose and mannose metabolism, aligning with the modest taxonomic shifts observed in rare taxa such as Eggerthellaceae, which possess specialized carbohydrate-processing enzymes [28,64]. These findings suggest cocoa polyphenols modulate a narrow set of microbial functions rather than inducing broad functional restructuring.

The basal diet exerted a broad influence on the gut microbiome, particularly on species evenness, which was lower in mice fed a Western-type diet. This effect is consistent with prior reports linking high-fat, high-sugar diets to disrupted microbial balance [40,65]. Interestingly, the microbiome’s response to cocoa supplementation varied depending on the background diet, suggesting that the effects of dietary polyphenols may depend on the broader nutritional context. This interaction mirrors findings from other polyphenol studies, such as those involving black raspberry supplementation in colitis models [41]. In terms of overall community structure, diet had a dominant role, with cecal and fecal microbiomes clustering by dietary pattern. These findings reinforce the idea that while the basal diet drives large-scale shifts in microbial ecology, polyphenol-rich supplements, such as cocoa, may fine-tune specific taxa or functions rather than overhauling the entire microbial community.

Unlike the broad impact of basal diet, cocoa supplementation produced more targeted shifts in the gut microbiome. These effects were most evident in the cecal and fecal communities, where cocoa altered microbial composition and appeared to promote the presence of rare taxa. This observation aligns with prior studies reporting enrichment of bacteria families, such as Lactobacillaceae, Bifidobacteriaceae, and Flavobacteriaceae, following cocoa intake [28,30,66,67]. Notably, our findings diverge from studies using higher cocoa doses in different models, which reported reduced microbial diversity [67], suggesting that both dosage and disease context shape outcomes. The more moderate cocoa dose used here may be more translatable to human consumption and still sufficient to influence specific microbial populations. These results support the idea that while basal diet induces community-wide changes in the microbiome, polyphenol-rich supplements can fine-tune composition by promoting specific, potentially beneficial taxa.

Among the rare taxa enriched by cocoa supplementation was the family Monoglobaceae, a member of the Clostridia class. Although little is known about its functional role, this family has been observed in other models involving dietary polyphenols, such as chia flour supplementation [68,69]. The increase in Monoglobaceae abundance in response to cocoa suggests a potential association between specific polyphenol compounds and the proliferation of low-abundance taxa. However, further research is needed to understand the biological significance of these shifts.

This study has several limitations. Our analysis focused exclusively on bacterial communities using 16S rRNA sequencing, which does not detect viral or eukaryotic components of the gut microbiome. Additionally, this approach provides only relative abundance data; absolute bacterial counts were not measured and may vary across different gut regions. A substantial proportion of sequences, particularly in mucosal samples, could not be taxonomically classified, limiting our understanding of key contributors in that niche. Functional pathway predictions based on tax4fun2 rely on inferred gene content and do not reflect direct microbial activity. Incorporating metabolomics in future studies would help confirm these predicted functional profiles and clarify microbial contributions to host metabolism. Another limitation is the use of only one cocoa polyphenol concentration in the diet. While this dose was chosen to reflect a realistic dietary supplement level, it is possible that different doses or dosing frequencies could elicit more substantial or more sustained effects. Finally, only male mice were used in this study, so potential sex-specific microbiome responses to diet, inflammation, or supplementation were not assessed.

## 5. Conclusions

In conclusion, this study demonstrates that cocoa polyphenol supplementation modulates the gut microbiome in a site-specific and time-dependent manner within a murine model of colitis and Western-style diet. Cocoa intake was associated with increased microbial diversity and the subtle enrichment of specific taxa in the cecal and fecal microbiomes, particularly before colitis induction, but had a limited impact on the mucosal microbial community. These findings emphasize the importance of examining multiple gastrointestinal sites when evaluating dietary interventions, as reliance on fecal samples alone may overlook important niche-specific responses. While cocoa polyphenols did not mitigate inflammation in this model, their selective influence on microbiome composition suggests that further investigation is warranted into how polyphenol metabolism and microbial function interact in the lower gut. Future studies should explore the fate of unclassified taxa in the mucosa, the durability of microbiome shifts with different dosing strategies, and the translational potential of these findings in human dietary contexts. Incorporating metabolomic and functional host data will be crucial to validate predicted pathways and establish a link between microbial changes and gut health outcomes.

## Data Availability

Supporting sequencing data for this manuscript are available to the public at the Utah State University Digital Commons repository, https://digitalcommons.usu.edu/all_datasets/246 (deposited on 11 July 2025). Available files include the .txt mapping file with sample attribute information, .csv file with 16S rRNA sequence count data with ASV identifiers, the .csv file with taxonomy mapped to the ASV identifier, and the phylogenetic tree file. All other data are contained within the article or the accompanying Supplementary Materials.

## Supplementary Materials

The following supporting information can be downloaded at: https://www.mdpi.com/article/10.3390/nu17172876/s1, Figure S1: Assessment of not assigned ASVs on beta diversity analyses; Figure S2: Rarefaction curve analysis grouped by time point and site; Figure S3: Relative abundance of murine intestinal bacterial taxa; Figure S4: Main effects of experimental site, time point, diet, and supplement on bacteria phyla relative abundance; Figure S5: Relative abundance of bacteria phyla for sample site within each time point or time point within each sample site; Figure S6: Relative abundance of bacteria phyla diet and supplement within each experimental sample site and time point; Figure S7: Main effects of sample site or time point on bacteria family relative abundance; Figure S8: Relative abundance of bacteria families for sample site within each time point; Figure S9: Relative abundance of bacteria families for time point within each sample site; Figure S10: Main effects of (a) diet or (b) supplement on relative abundance of select bacteria families; Figure S11: Effect of CP supplementation on the relative abundance of select bacteria families; Figure S12: Bacillota:Bacteroidota (B:B) ratio (formerly Firmicutes:Bacteroidetes); Figure S13: Alpha diversity of microbiomes considering the main effects of site, time point, diet, and supplement for observed ASVs and the Shannon diversity index; Figure S14: Beta diversity plots for main effects of site, time point, diet, and supplement; Figure S15: Beta diversity plots for effects time point within each sample site and effects of site within each time point.; Figure S16: Predicted functional capacity of gut microbiomes for sample site pairwise comparisons; Figure S17: Predicted functional capacity of gut microbiomes for time point pairwise comparisons; Figure S18. Predicted functional capacity of gut microbiomes for basal diet or supplement pairwise comparisons; Table S1: Experimental diet formulations; Table S2: Effects of diet and supplement on Bacillota:Bacteroidota ratio within each sample site and time point; Table S3: Pairwise comparisons of diet × supplement on Bacillota: Bacteroidota ratio for each time point and sample site; Table S4: Main effects of time point, site, diet and supplement on microbiome alpha diversity; Table S5: Effects of diet and supplement on alpha diversity within each sample site and time point; Table S6: Pairwise comparisons of diet × supplement on alpha diversity for each time point and sample site; Table S7: Effects of diet and supplement at each sample site and time point on unweighted and weighted UniFrac distance beta diversity; File S1: Normalized sequence counts.csv; File S2: MaAsLin2 multivariate analyses results.xlsx; File S3: Pathway enrichment analyses results.xlsx.
