# Supplementary material for: Cocoa Polyphenols Modulate the Mouse Gut Microbiome in a Site-Specific Manner
Source: Nutrients. 2025 Sep 5;17(17):2876. doi: 10.3390/nu17172876 (PMC12430346; doi:10.3390/nu17172876)
Supplement: Supplementary file 1 [file nutrients-17-02876-s001.zip › Hayden_Nutrients_supplementary material.pdf]

nutrients

MDPI

Supplementary Material

Cocoa Polyphenols Modulate the Mouse Gut Microbiome in a Site-Specific Manner

Marcus Hayden, Eliza C Stewart, Mohammed Almatani, Jeremy Case, Samuel Rice, Giovanni Rompato, Korry Hintze, and Abby Benninghoff*

* Correspondence: abby.benninghoff@usu.edu; Tel.: +01-435-797-8649

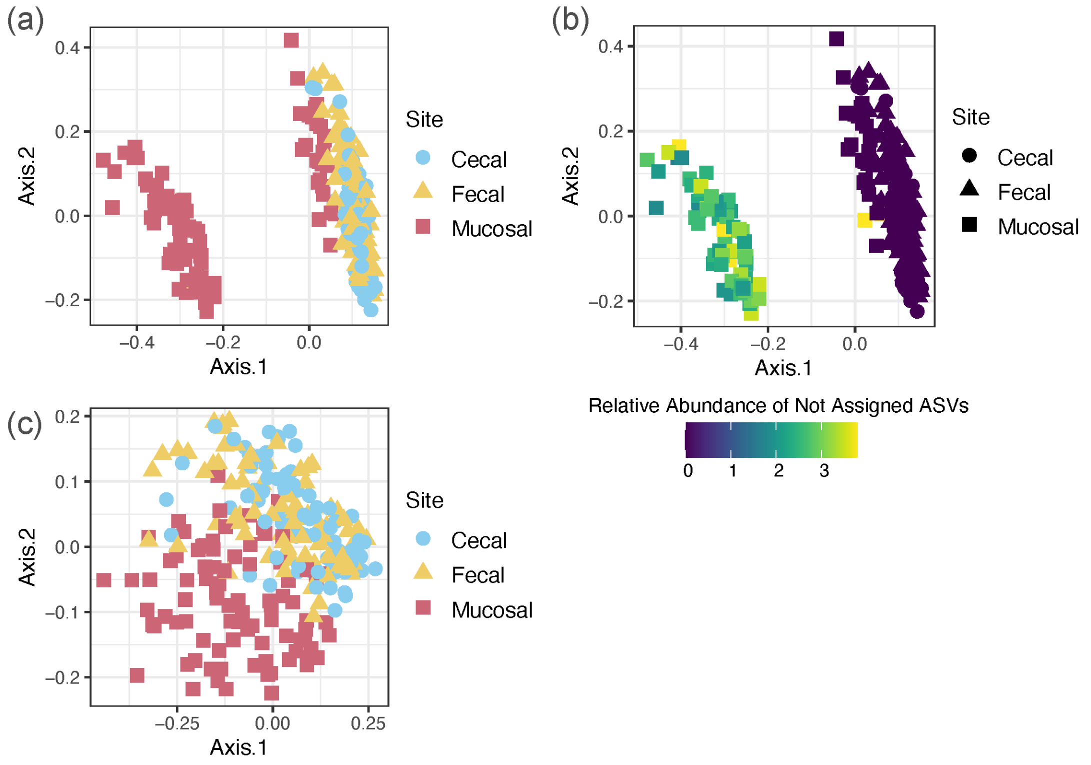

Figure S1. Assessment of *not assigned* ASVs on beta diversity analyses. (**a**) Unweighted UniFrac beta diversity principal coordinate analysis for all ASVs organized by sample site. (**b**) Data are the same as panel (a) but were colored by the normalized relative abundance of ASVs designated as *not assigned*. Note, these sequences are nearly exclusive to samples from colon mucosa. (**c**) Unweighted UniFrac beta diversity PCoA excluding *not assigned* ASVs.

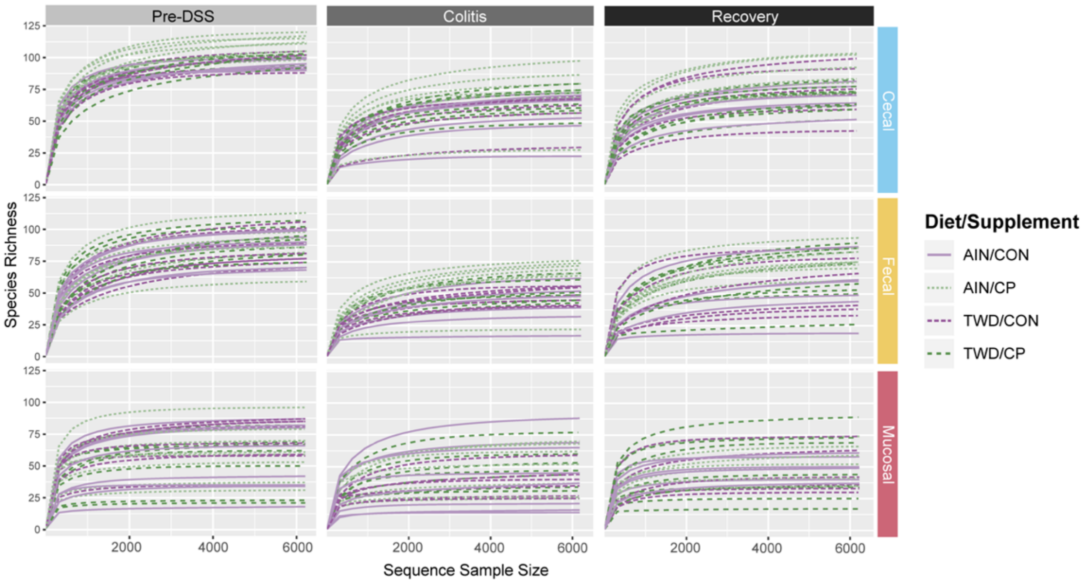

Figure S2. Rarefaction curve analysis grouped by time point and site. Diet and supplement groups are indicated by line style. The sequence sample size for the samples of this study is sufficient to discern the maximum amount of sequence information. The rarefaction plots show how species richness changes as sequence sample size increases. Rarefaction plots are used to assess if the sequencing depth is sufficient to discern the entirety of the microbiome within the sample. Data were rarefied to the lowest total among all the samples: ~6000 sequences.

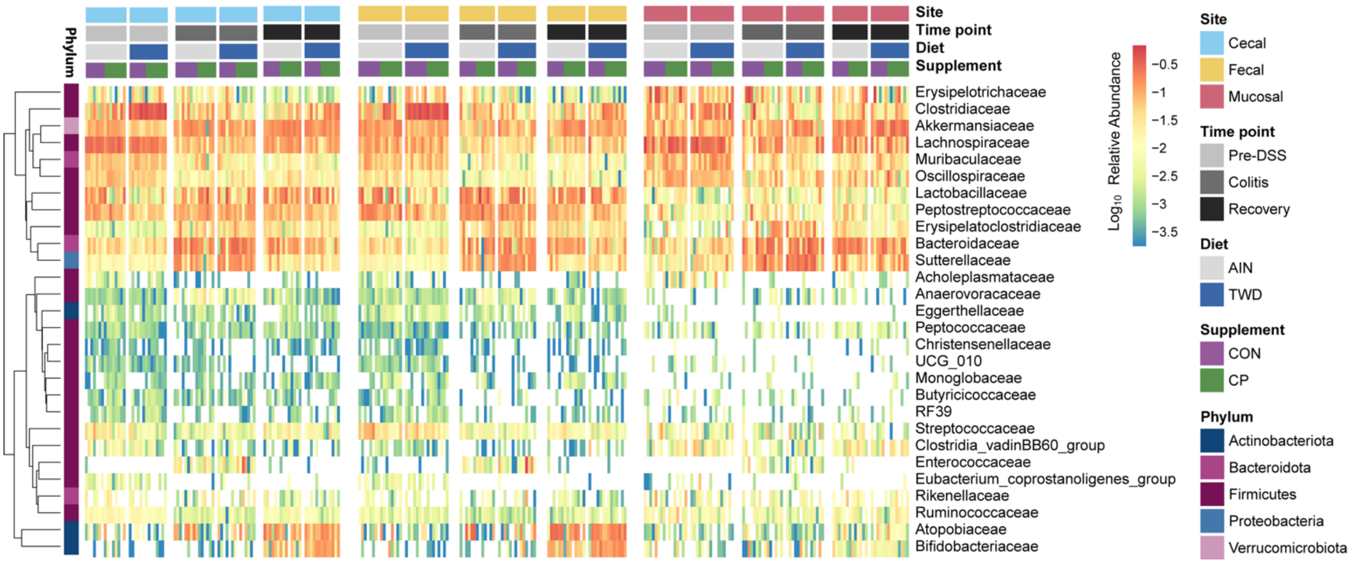

Figure S3. Relative abundance of murine intestinal bacterial taxa. An unsupervised hierarchical cluster analysis was used to illustrate the relative abundance at the family level. Colors within the figure correspond to $\log_{10}$ relative abundance of the family using the Euclidean distance with average linkage (n = 6 to 8 mice per experimental subgroup). White space within the heatmap indicates that the family was not detected in the sample.

Microbiome Taxonomic Composition - Phyla

Significant differences in bacterial phyla were observed across experimental factors, with timepoint emerging as the primary driver of compositional change (Figures 3 and S4; File S2). All phyla except Verrucomicrobiota differed significantly between pre-DSS and colitis, with Firmicutes ($p = 2.02\times10^{-17}$), Proteobacteria ($p = 3.54\times10^{-25}$), and Actinobacteriota ($p = 3.56\times10^{-2}$) showing marked shifts (Figure S3). Notably, Actinobacteriota, Firmicutes, Proteobacteria, and Verrucomicrobiota also differed significantly between pre-DSS and recovery ($p < 10^{-5}$), suggesting that the baseline microbiome was not fully restored by the recovery time point. Site-specific differences were most pronounced for Actinobacteriota, Bacteroidota, and Proteobacteria, with significant differences between mucosal samples and both cecal and fecal sites (e.g., Actinobacteriota, cecal vs. mucosal: $p = 1.08\times10^{-4}$; fecal vs. mucosal: $p = 1.36\times10^{-6}$) (Figure S4). In contrast, Verrucomicrobiota showed no site-dependent differences. Dietary effects were limited, with only Proteobacteria differing significantly between the AIN and TWD diets ($p = 5.26\times10^{-3}$) (Figure S3d). CP supplementation did not significantly alter phylum-level abundance for any group examined. These findings highlight the dominant influence of inflammation and recovery on microbial community structure, with more modest or localized effects of diet and supplementation.

To explore context-specific effects of diet and CP supplementation, we performed pairwise analyses within each site and timepoint (Figure S5). Firmicutes and Verrucomicrobiota were significantly affected by diet in the cecal microbiome at the pre-DSS timepoint ($p = 3.17\times10^{-3}$ for both phyla), despite minimal effects observed in the main model. At colitis, Firmicutes also showed significant dietary differences in the fecal compartment ($p = 4.00\times10^{-4}$), and Verrucomicrobiota was significantly altered in both fecal ($p = 2.92\times10^{-2}$) and mucosal samples ($p = 3.80\times10^{-3}$). Proteobacteria exhibited a diet-related difference in fecal samples during colitis ($p = 3.83\times10^{-2}$), aligning with the increased abundance observed under inflammatory conditions. While CP supplementation did not show consistent effects across conditions, significant differences were observed for Actinobacteriota in fecal samples at Pre-DSS in AIN-fed mice, and for Verrucomicrobiota in feces under multiple conditions: increased with CP in TWD-fed mice at pre-DSS, and elevated in AIN-fed mice at recovery, with a significant main effect at this timepoint.

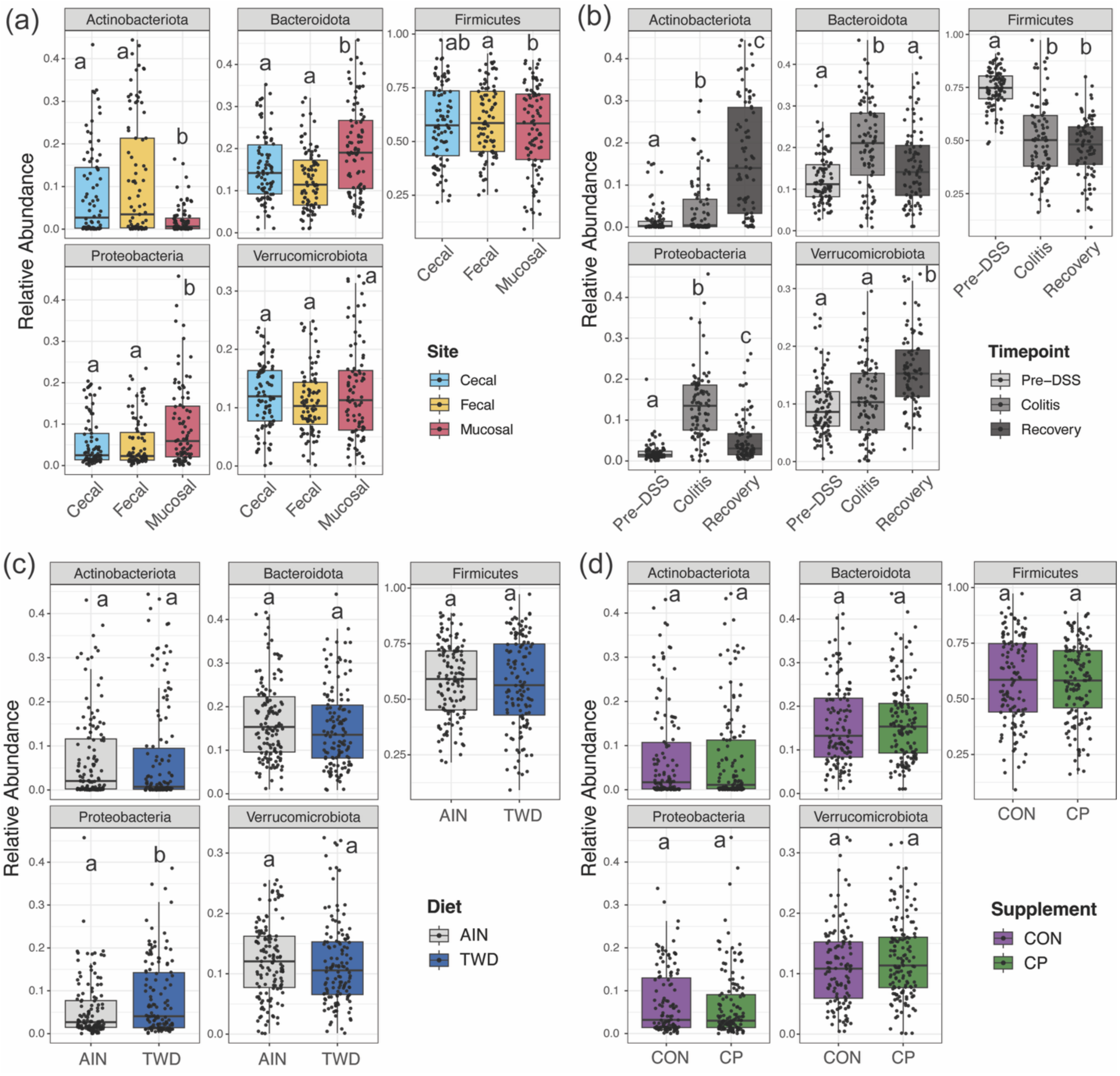

Figure S4. Main effects of experimental site, time point, diet, and supplement on bacteria phyla relative abundance. Data are shown as Tukey box-and-whisker plots for relative abundance of each phylum for (**a**) microbiome site (n = 86 to 88), (**b**) time point (n = 83 to 93), (**c**) basal diet (n = 130 to 131), and (**d**) supplement group (n = 122 to 139). Different letters indicate that the relative abundances among groups within the plot are statistically different (FDR $p < 0.05$). Complete results of MaAsLan2 multivariate analyses are provided in File S2.

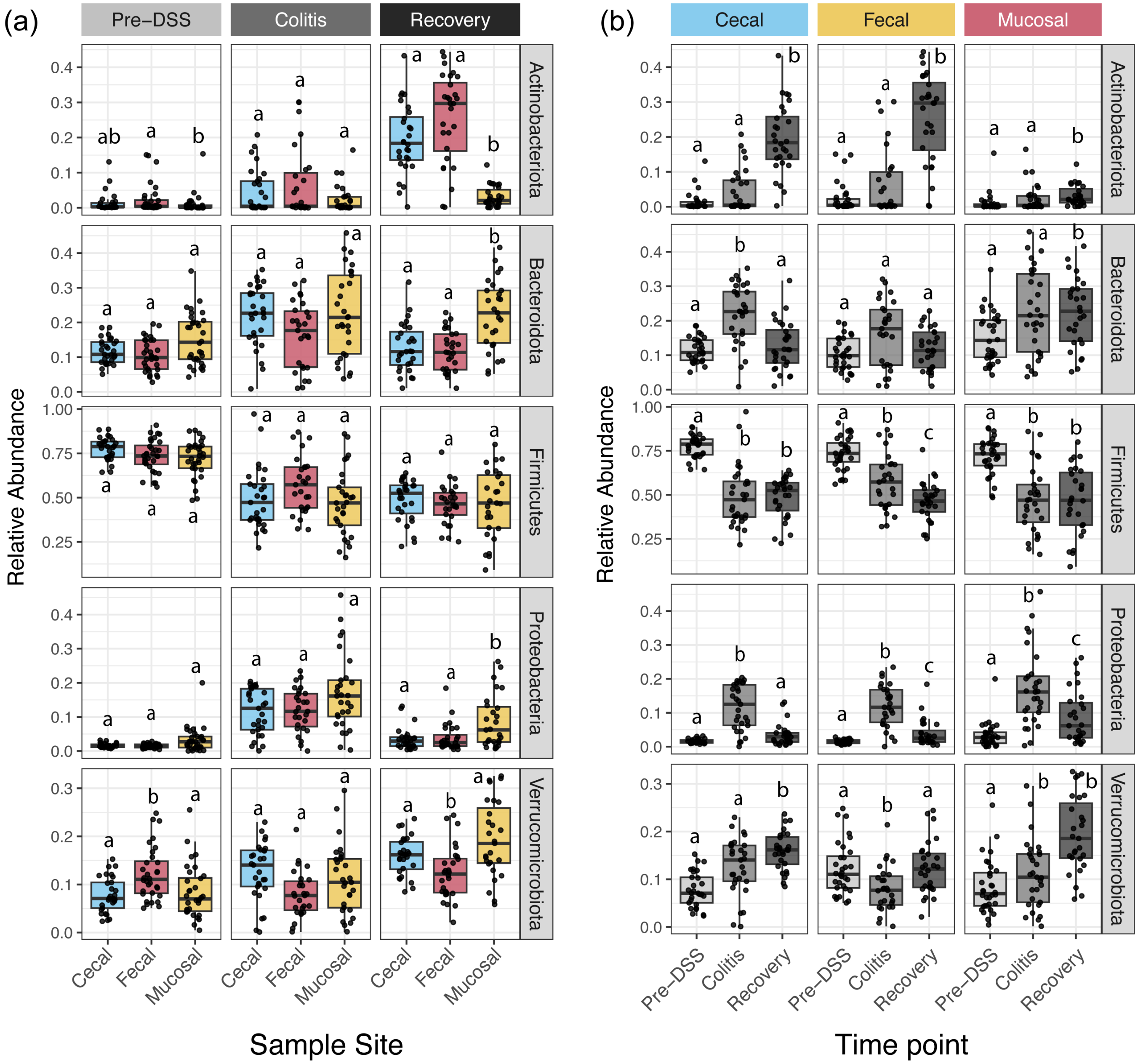

Figure S5. Relative abundance of bacteria phyla for (**a**) sample site within each time point or (**b**) time point within each sample site. Data are shown as Tukey box-whisker plots for the relative abundance of Actinobacteriota, Bacteroidota, Firmicutes, Proteobacteria, and Verrucomicrobiota phyla (n = 27 to 32 for each site × time point subgroup). Different letters indicate that the relative abundances among groups within the plot are statistically different (FDR p<0.05). Complete results of MaAsLan2 multivariate analyses are provided in File S2.

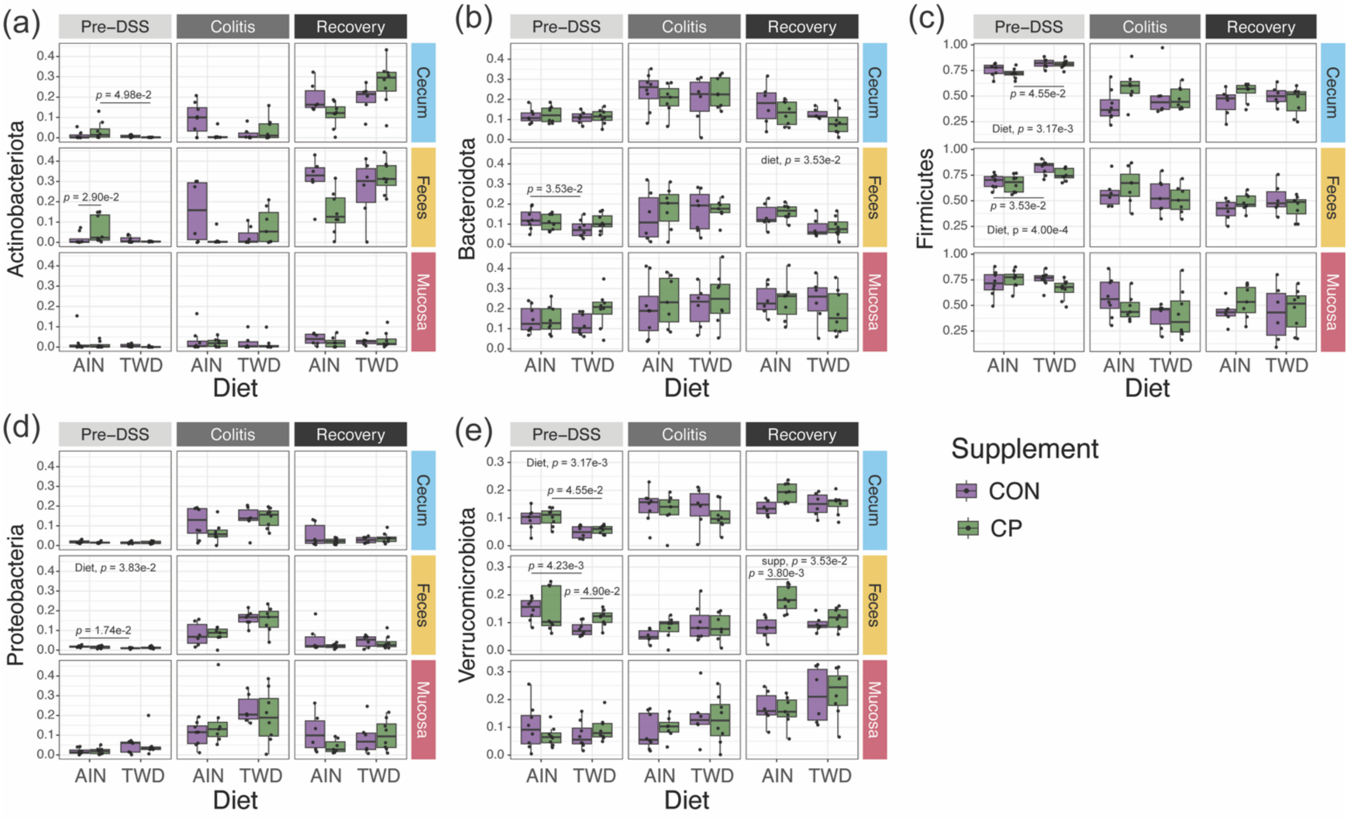

Figure S6. Relative abundance of bacteria phyla diet and supplement within each experimental sample site and time point. Data are shown as Tukey box-and-whisker plots for the relative abundance of (**a**) Actinobacteriota, (**b**) Bacteroidota, (**c**) Firmicutes, (**d**) Proteobacteria, and (**e**) Verrucomicrobiota phyla (n = 6 to 8 for each diet × supplement experimental subgroup). Significant differences between CON and CP supplement groups are noted within the graph, as well as main effects of diet or supplement within each site and time point subgroup. Complete results of MaAsLan2 multivariate analyses are provided in File S2.

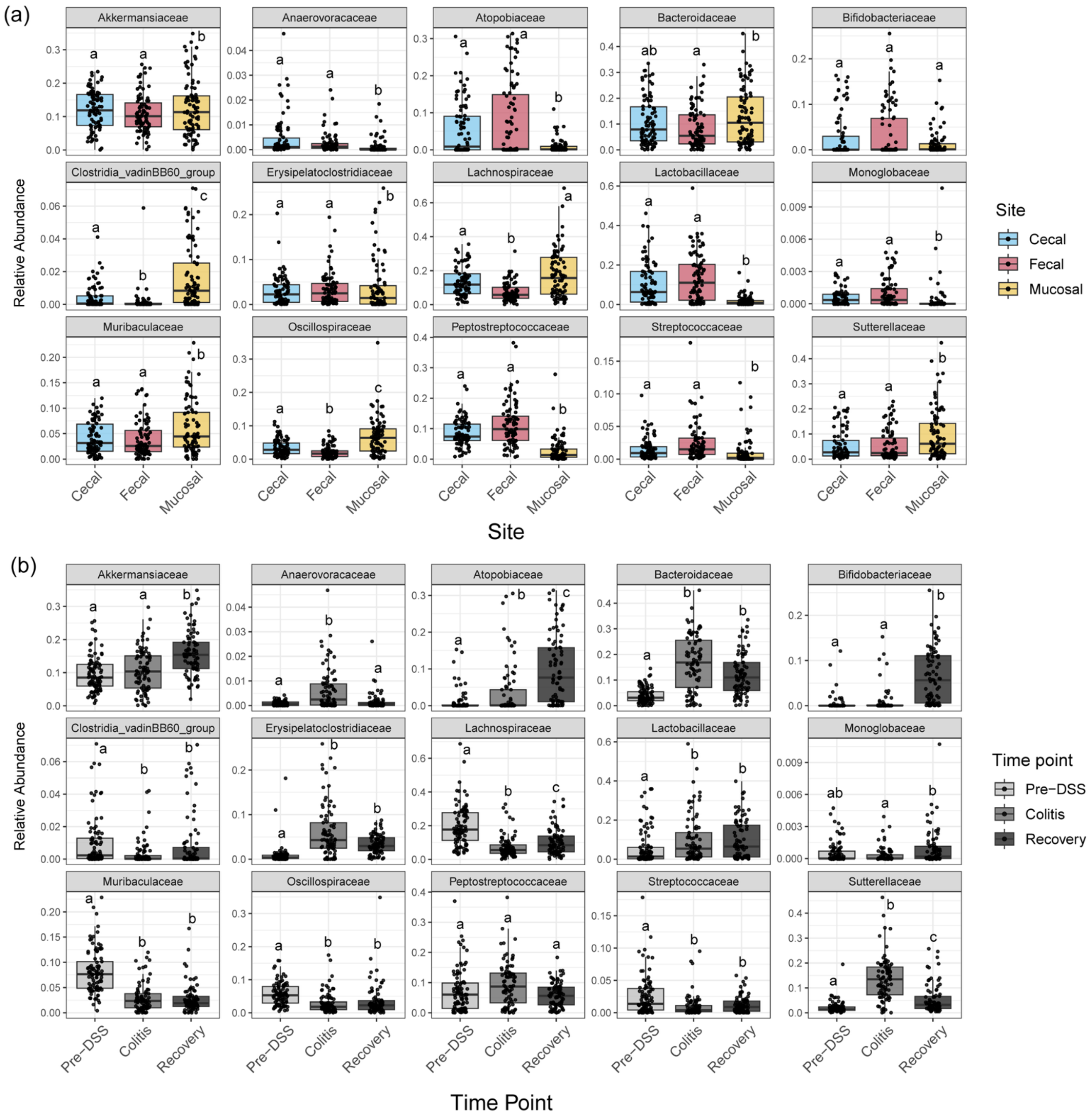

Figure S7. Main effects of (**a**) sample site or (**b**) time point on bacteria family relative abundance. Data are shown as Tukey box-and-whisker plots for relative abundance for selected bacteria families for sample site (n = 86 to 88) or time point (n = 83 to 93). Different letters indicate that the relative abundances among groups within the plot are statistically different (FDR $p < 0.05$). Complete results of MaAsLan2 multivariate analyses are provided in File S2.

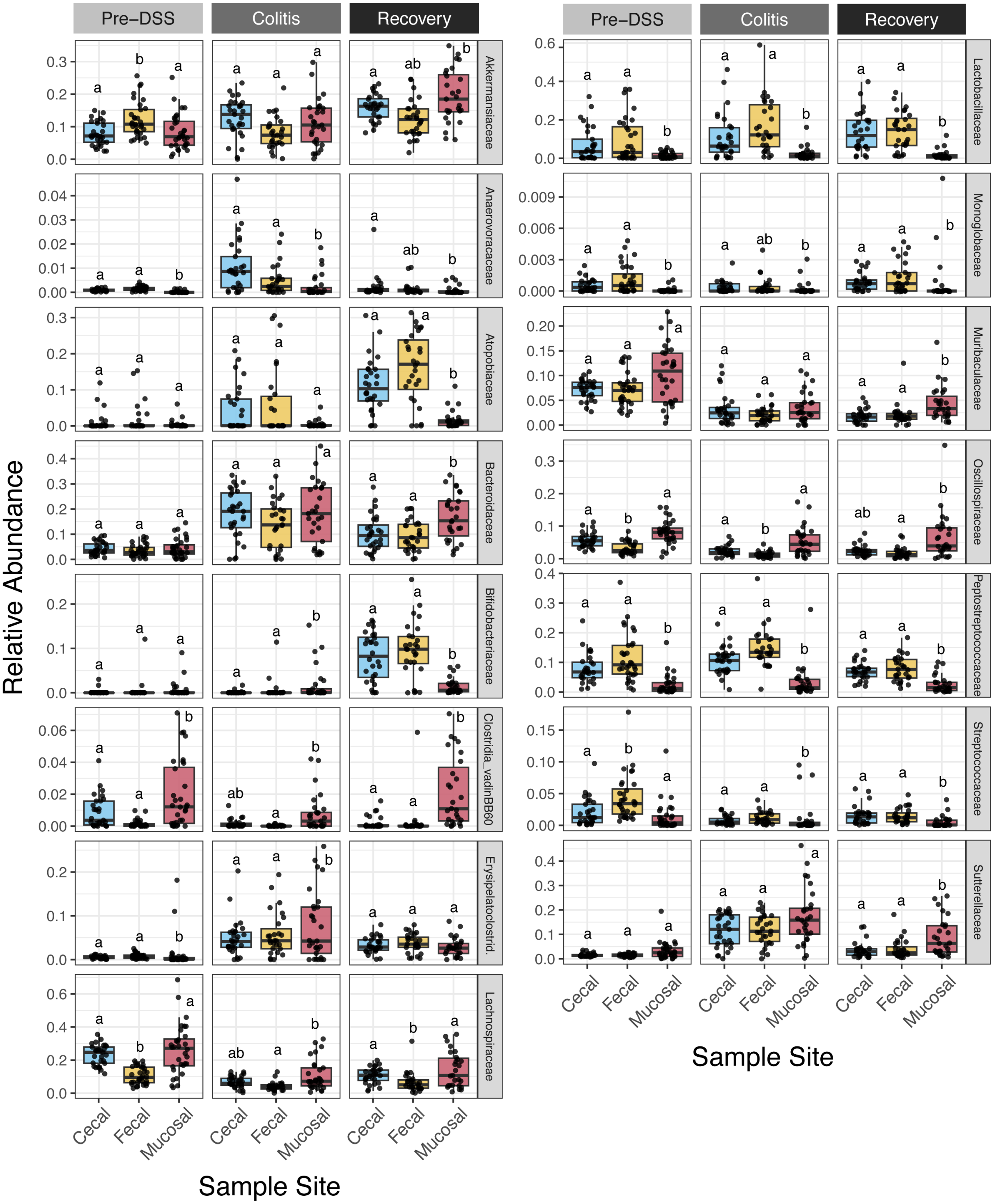

Figure S8. Relative abundance of bacteria families for sample site within each time point. Data are shown as Tukey box-and-whisker plots for the relative abundance of select bacteria families of interest (n = 27 to 32 for site × time point subgroups). Different letters indicate that the relative abundances among groups within the subplots are statistically different (FDR $p < 0.05$). Complete results of MaAsLan2 multivariate analyses are provided in File S2.

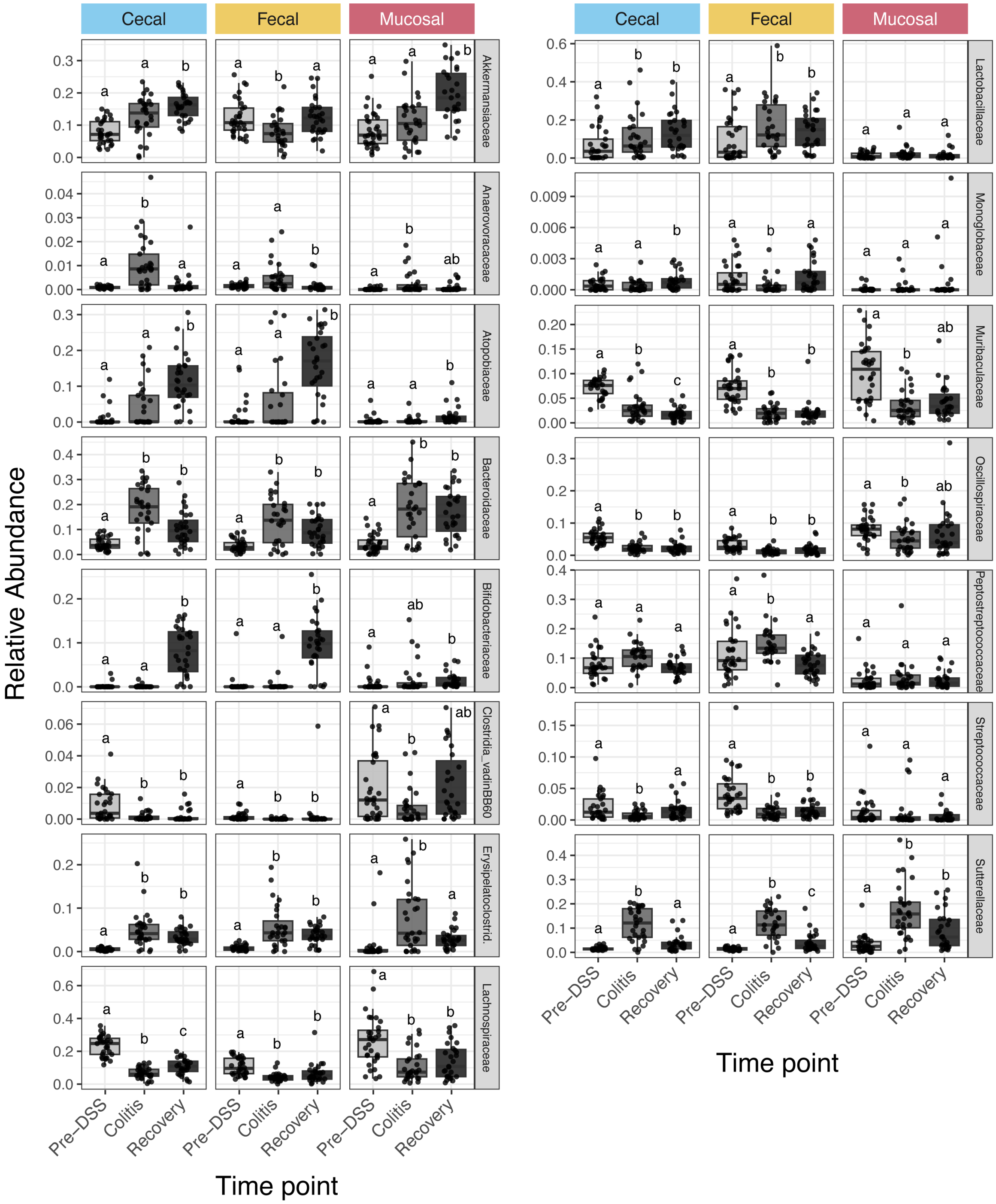

Figure S9. Relative abundance of bacteria families for time point within each sample site. Data are shown as Tukey box-and-whisker plots for the relative abundance of select bacteria families of interest (n = 27 to 32 for site × time point subgroups). Different letters indicate that the relative abundances among groups within the subplots are statistically different (FDR $p < 0.05$). Complete results of MaAsLan2 multivariate analyses are provided in File S2.

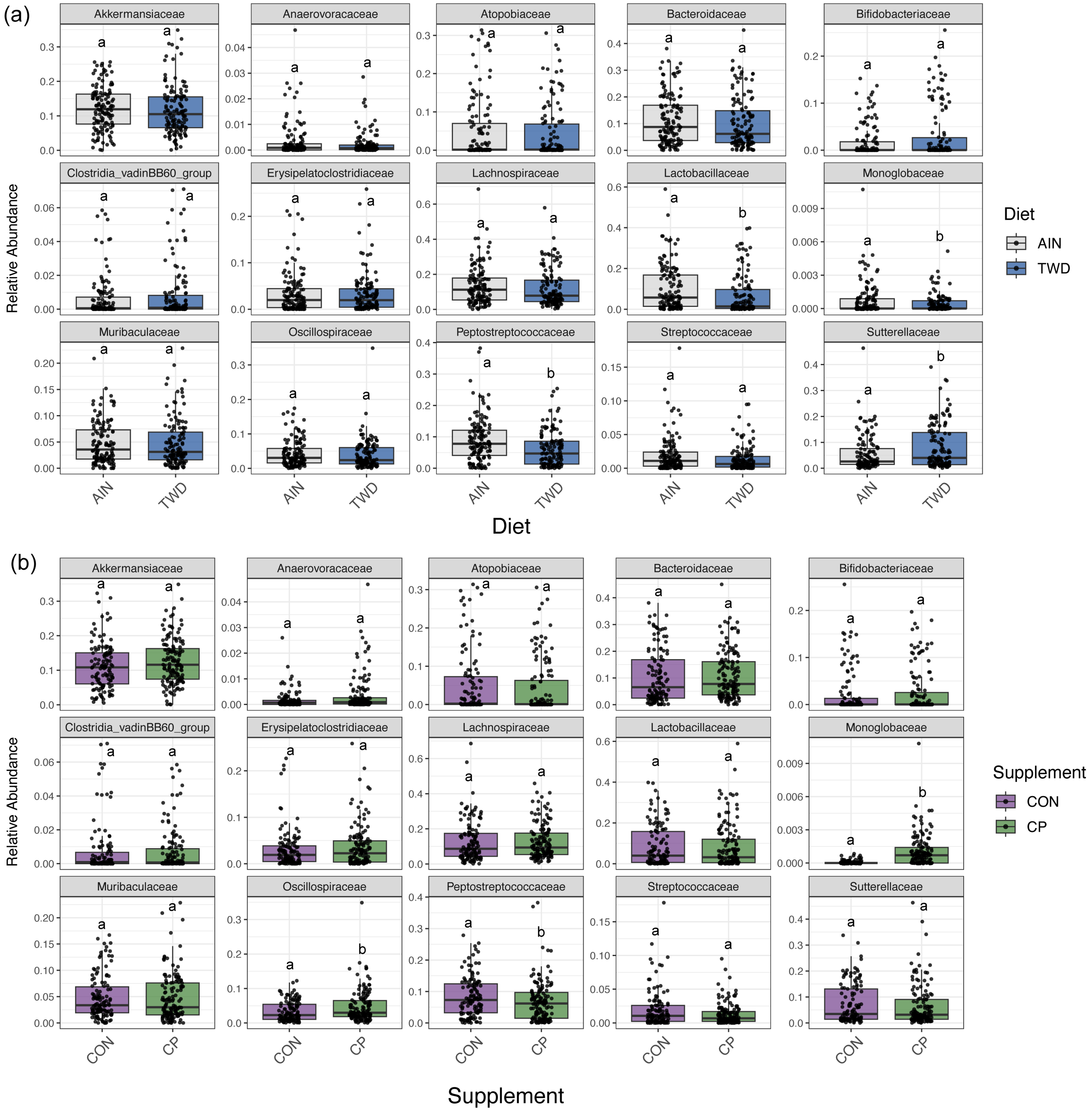

Figure S10. Main effects of (**a**) diet or (**b**) supplement on bacteria phyla relative abundance. Data are shown as Tukey box-and-whisker plots for relative abundance of selected bacterial families for basal diet (n = 130 to 131) or supplement group (n = 122 to 139). Different letters indicate that the relative abundances among groups within the plot are statistically different (FDR $p < 0.05$). Complete results of MaAsLan2 multivariate analyses are provided in File S2.

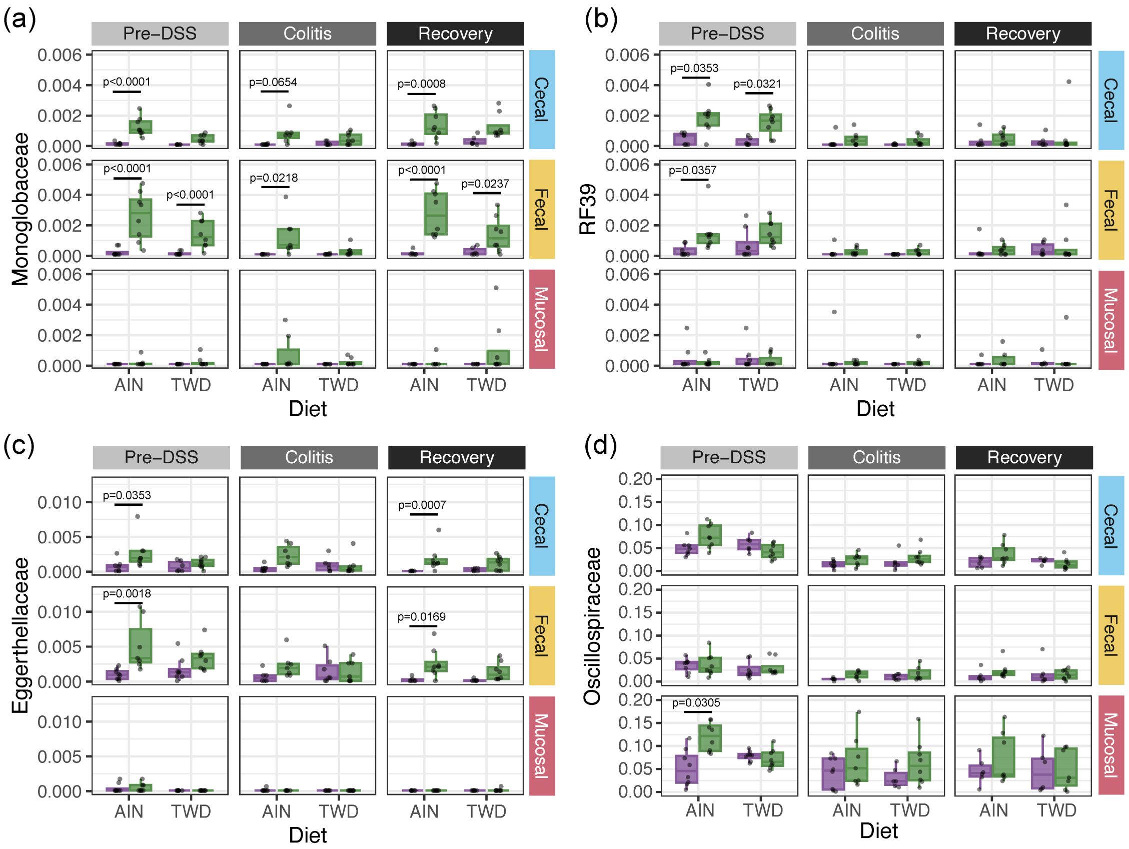

Figure S11. Effect of CP supplementation on the relative abundance of select bacteria families. Relative abundance values for (**a**) Monoglobaceae, (**b**) RF39 (order Bacilli), (**c**) Eggerthellaceae, and (**d**) Oscillospiraceae are shown. Significant effects of CP supplement are indicated where applicable as determined by MaAsLin2 analysis within each time point and site; complete statistical results are available in File S2. Abbreviations include TP, time point; Supp, Supplement; AIN, AIN93 basal diet; TWD, total Western diet; CON, control; CP, coca polyphenol.

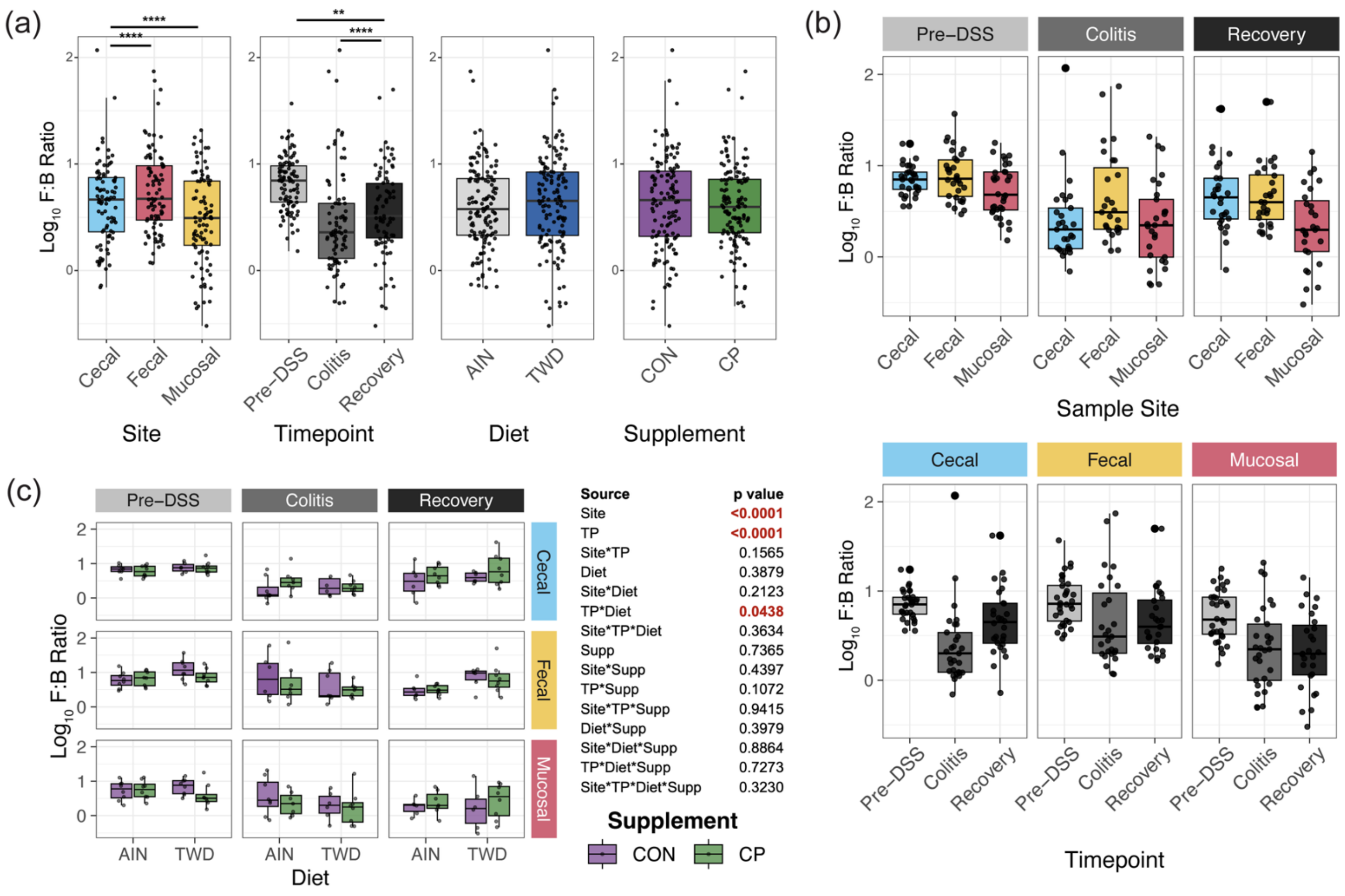

Figure S12. Firmicutes:Bacteroidota (F:B) ratio. (**a**) Main effects of site, time point, diet, and supplement on the $\log_{10}$ F:B ratio shown as Tukey box-and-whisker plots. **, $p < 0.01$, ****, $p < 0.0001$ as determined by a generalized linear model (with Tukey HSD post-hoc test as appropriate) as described in Materials and Methods (site, n = 86 to 88; time point, n = 83 to 93; basal diet, n = 130 to 131; cocoa polyphenol supplement, n = 122 to 139). (**b**) Effect of sample site within each time point (top) or of time point within each sample site (bottom) on the $\log_{10}$ F:B ratio shown as Tukey box-and-whisker plots (n = 27 to 32 within each site × time point subgroup). Different letters indicate that the compared groups within each subplot are statistically different. (**c**) Effect of diet and supplement within each site and time point shown as Tukey box-and-whisker plots (n = 6 to 8 within each diet × supplement subgroup). The inset table provides the overall main effects for each experimental factor and all interactions as determined by general linear model analysis (see Materials and Methods). No significant differences among the diet and supplement groups were identified.

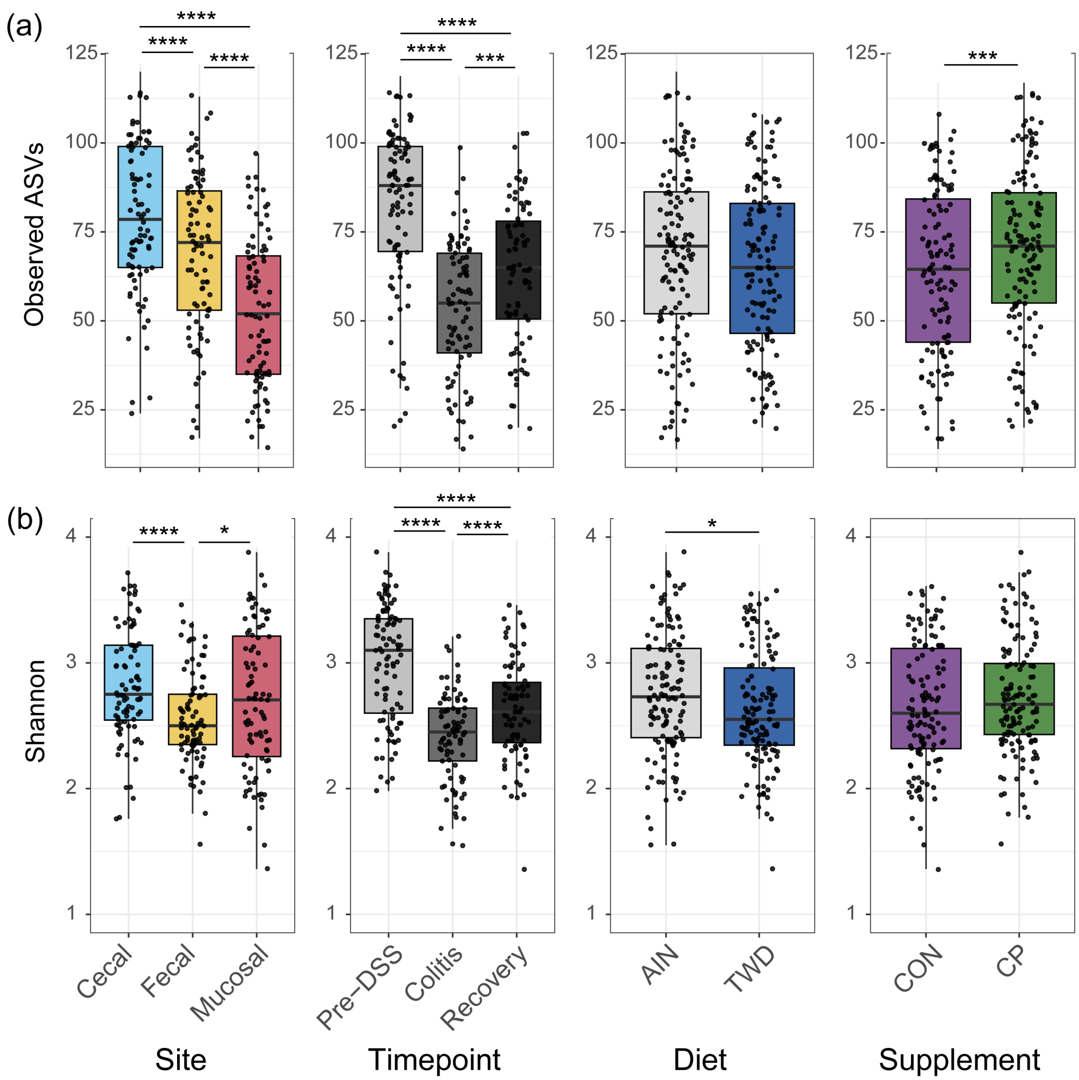

Figure S13. Alpha diversity of microbiomes considering the main effects of site, timepoint, diet, and supplement for (**a**) observed ASVs and (**b**) the Shannon diversity index. Data are shown as Tukey box-and-whisker plots with *, $p < 0.05$; ***, $p < 0.001$, ****, $p < 0.0001$ as determined by a generalized linear model (with Tukey HSD post-hoc test as appropriate) for main effects of time point, site, diet or supplement as described in Materials and Methods (site, $n = 86$ to 88; time point, $n = 83$ to 93; basal diet, $n = 130$ to 131; cocoa polyphenol supplement, $n = 122$ to 139).

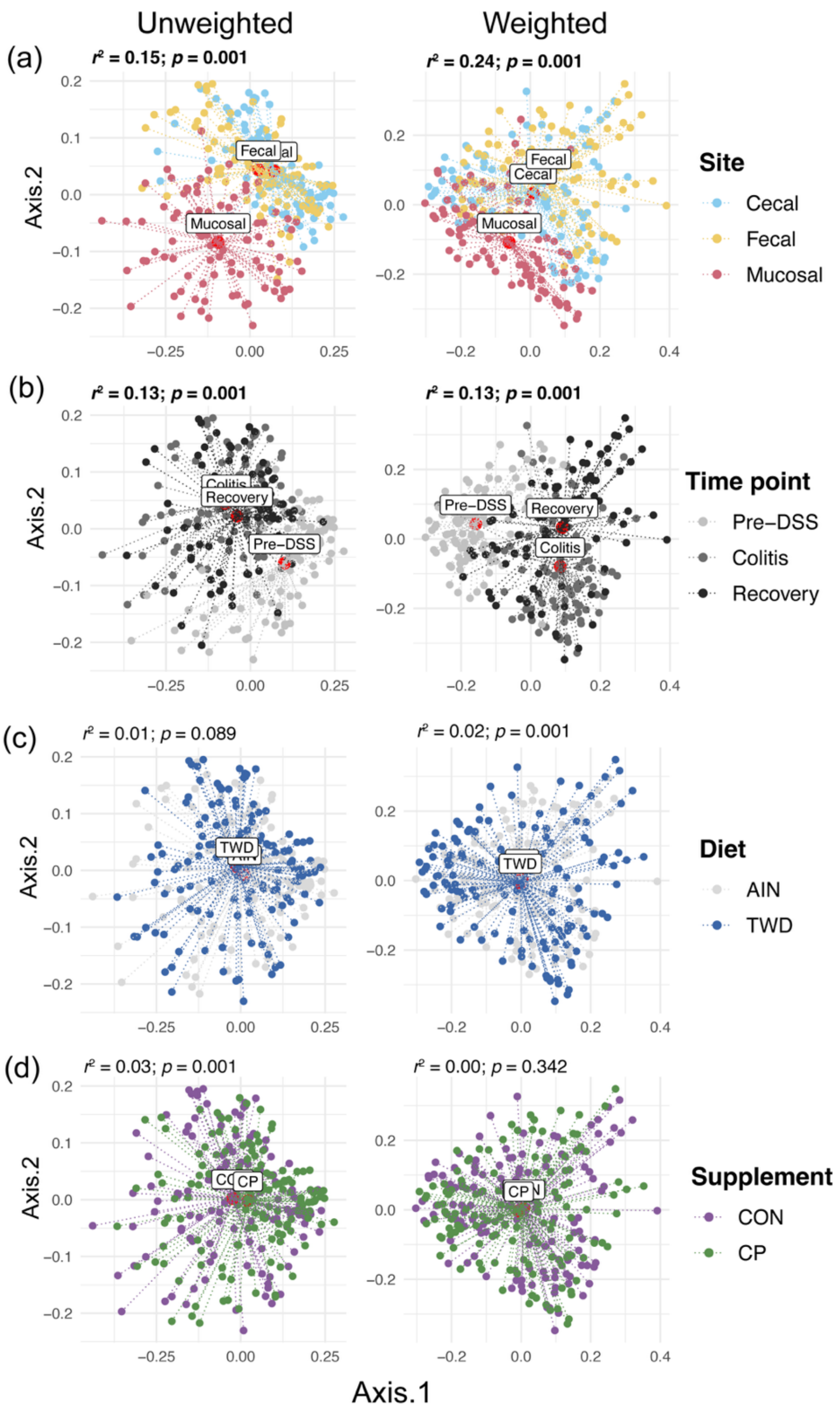

Figure S14. Beta diversity plots for main effects of site, time point, diet, and supplement. Data are shown as principal coordinate plots using the first two coordinates for (**a**) unweighted and (**b**) weighted UniFrac distances. The overall *R*-squared and PERMANOVA *p*-values for each plot are shown, with $R^2 > 0.1$ and $p < 0.05$ considered significant. For each plot, the centroid for each level within the main effect is shown (site, n = 86 to 88; time point, n = 83 to 93; basal diet, n = 130 to 131; cocoa polyphenol supplement, n = 122 to 139).

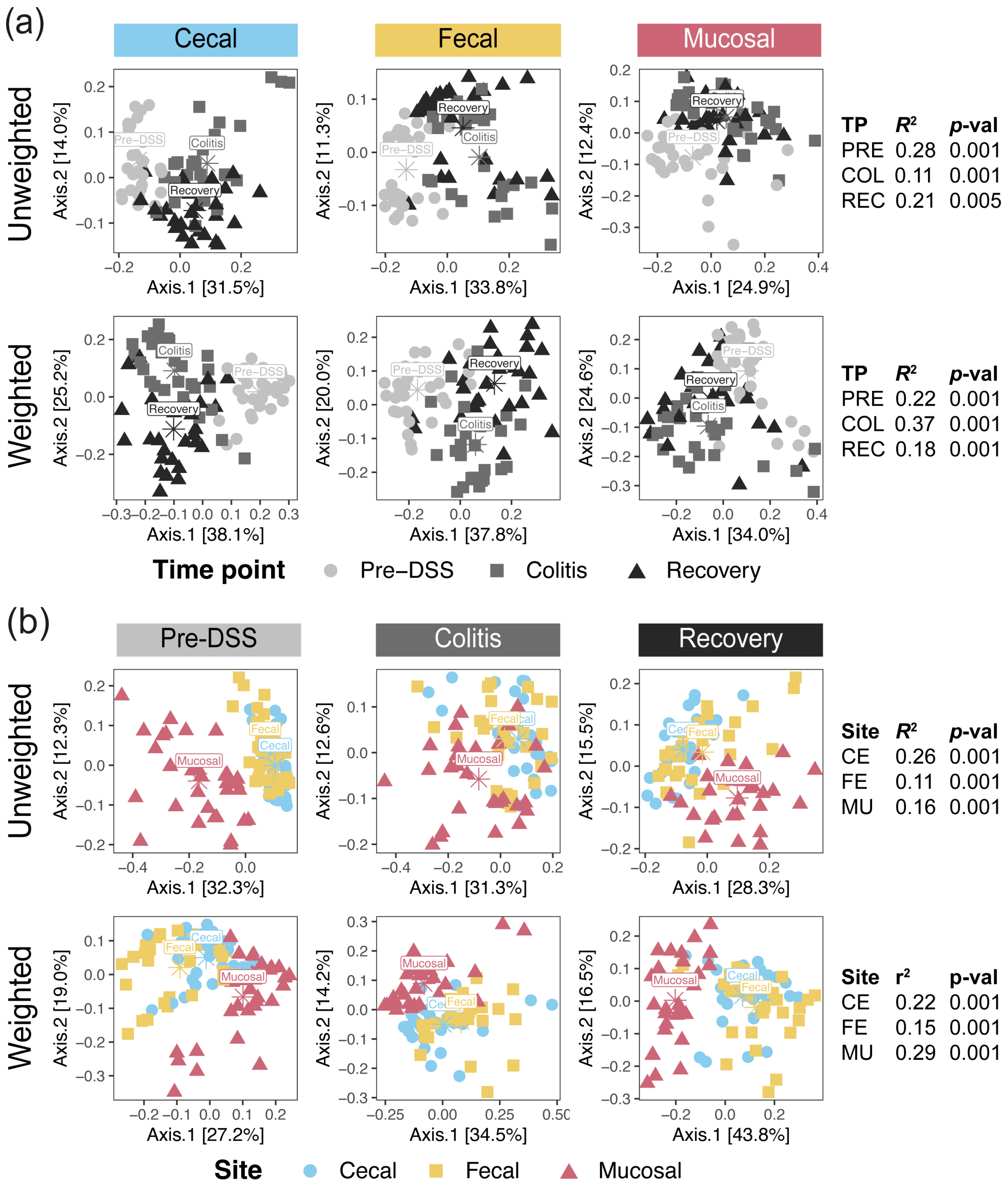

Figure S15. Beta diversity plots for (**a**) effects time point within each sample site and (**b**) effects of site within each time point. Data are shown as principal coordinate plots with centroids using the first two coordinates for unweighted and weighted UniFrac distances. The overall *R*-squared and PERMANOVA *p*-values for each plot are shown, with $R^2 > 0.1$ and $p < 0.05$ considered significant ($n = 27$ to 32 for each site × time point subgroup).

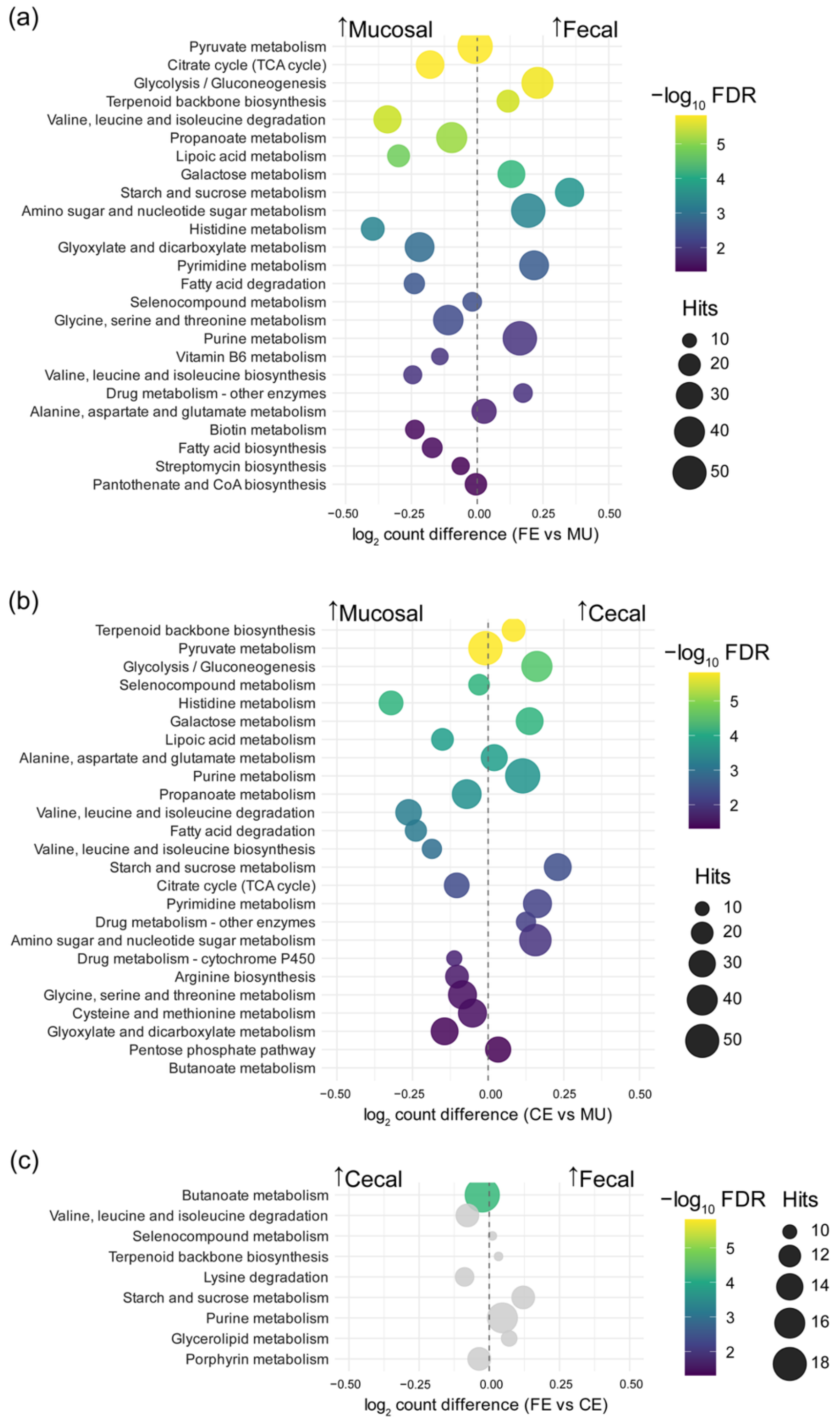

Figure S16. Predicted functional capacity of gut microbiomes for sample site pairwise comparisons. The bubble plots depict the -$\log_{10}$ FDR *p*-values and number of hits for each pathway following enrichment analysis of significant KEGG terms identified by MaAsLin2 multivariate analysis, while controlling for time point, diet, and supplement, as described in Materials and Methods (site, *n* = 86 to 88). Bubble position on the x-axis indicates the $\log_2$ difference of normalized KEGG term counts for pairwise comparisons for sample site. Complete results of the pathway enrichment analysis are available in File S3. Abbreviations include CE, cecal; FE, fecal; MU, mucosal.

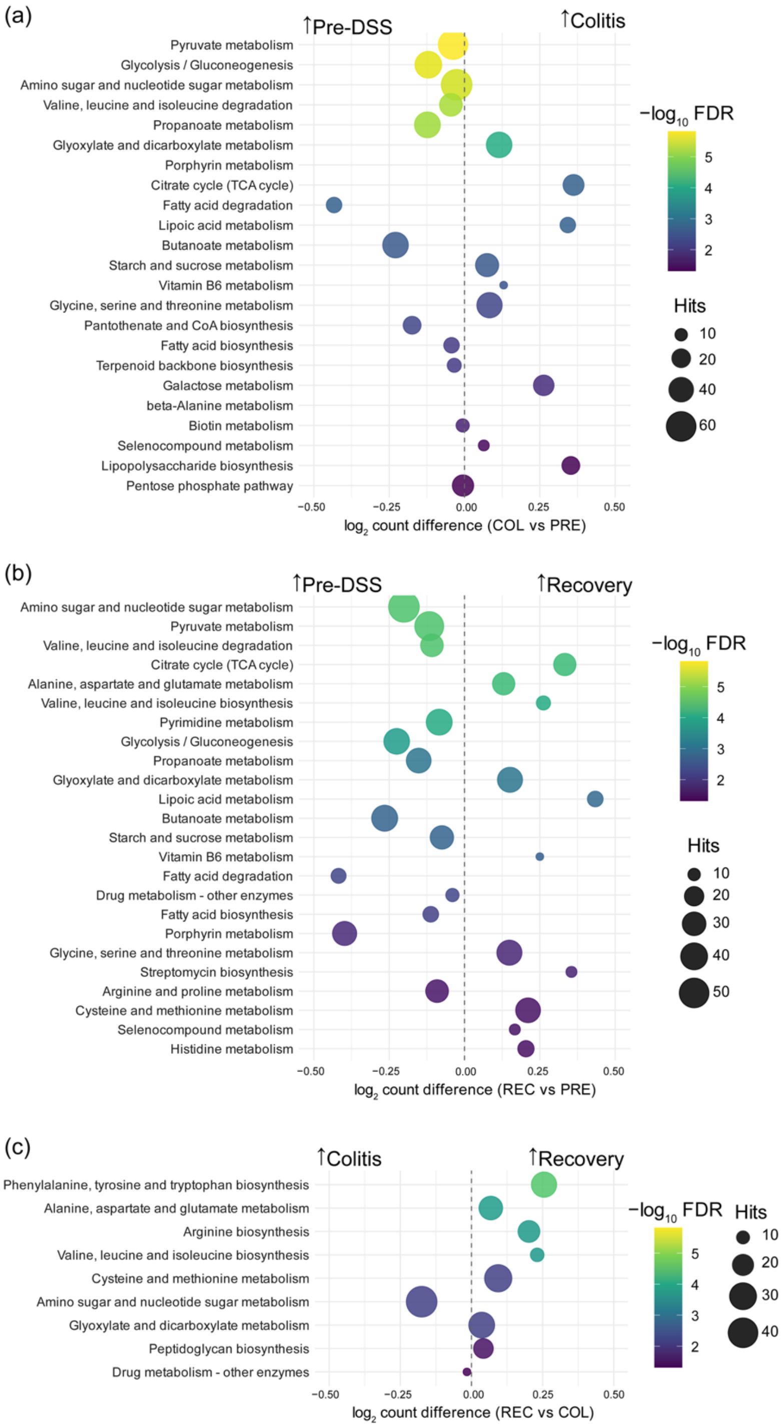

Figure S17. Predicted functional capacity of gut microbiomes for time point pairwise comparisons. The bubble plots depict the -$\log_{10}$ FDR p-values and number of hits for each pathway following enrichment analysis of significant KEGG terms identified by MaAsLin2 multivariate analysis, while controlling for site, diet, and supplement, as described in Materials and Methods (time point, n = 83 to 93). Bubble position on the x-axis indicates the $\log_2$ difference of normalized KEGG term counts between the each pairwise comparison for time point. Bubbles that are not colored indicate FDR p-values < 0.05 for pathway enrichment Complete results of the pathway enrichment analysis are available in File S3. Abbreviations include PRE, pre-DSS; COL, colitis; REC, recovery.

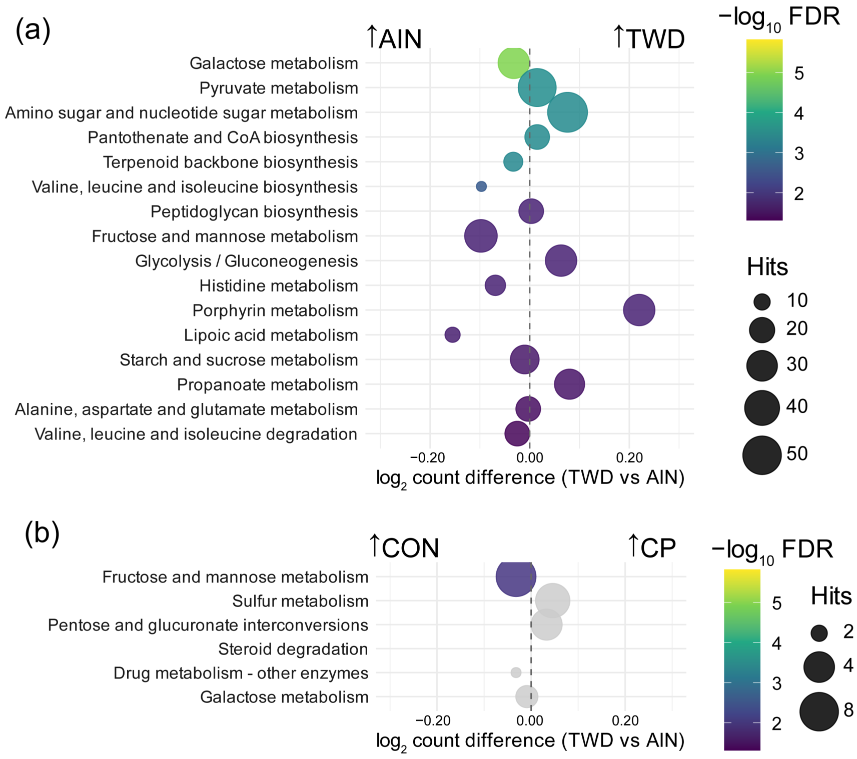

Figure S18. Predicted functional capacity of gut microbiomes for (a) basal diet or (b) supplement pairwise comparisons. The bubble plots depict the -log10 FDR *p*-values and number of hits for each pathway following enrichment analysis of significant KEGG terms identified by MaAsLin2 multivariate analysis, while controlling for site, time point, and either supplement (**a**) or diet (**b**), as described in Materials and Methods (diet, $n = 130$ to 131; supplement, $n = 122$ to 139). Bubble position on the x-axis indicates the $\log_2$ difference of normalized KEGG term counts between the experimental conditions compared. Bubbles that are not colored indicate FDR *p*-values < 0.05 for pathway enrichment. Complete results of the pathway enrichment analysis are available in File S3. Abbreviations include AIN, AIN93G diet; TWD, total Western diet; CON, control; CP, cocoa polyphenols.

Table S1. Experimental diet formulations.

	**AIN93G**	**AIN93G + CP**	**TWD**	**TWD + CP**
Energy density (kcal/g)	3.8	3.7	4.4	4.3
Carbohydrates (g/kg diet)				
Cocoa powder		25.6		25.6
Corn Starch	397.5	388.9	230	219.9
Maltodextrin	132	132	70	70
Sucrose	100	100	261.2	261.2
Cellulose	50	38	30	18
Kcal (% of total)	*63.9%*	*63.9%*	*50.0%*	*49.8%*
Proteins (g/kg)				
Casein	200	195	190	186.6
L-cystine	3	3	2.85	2.85
Kcal (% of total)	*18.8%*	*18.8%*	*15.5%*	*15.5%*
Fats (g/kg)				
Soybean oil	70	70	31.4	31.4
Anhydrous milk fat			36.3	36.3
Olive Oil			28.0	28.0
Lard			28.0	28.0
Beef tallow			24.8	24.8
Corn oil			16.5	16.5
Cholesterol			0.4	0.4
Kcal (% of total)	*17.2%*	*17.3%*	*34.5%*	*34.7%*
Mineral mix (35 g/kg)	AIN-93G-MX (94046)	AIN-93G-MX (94046)	nTWD (110422)	nTWD (110422)
Vitamin mix (10 g/kg)	AIN-93-VX (94047)	AIN-93-VX (94047)	nTWD (110423)	nTWD (110423)

Note: Abbreviations for diets are as follows: total Western diet, TWD; Cocoa polyphenols as CocoaVia™ Cardio Health Powder[1], CP. The composition of the TWD was published previously.[2] No data on chloride, manganese, iodine, pantothenic acid, biotin, or ultra-trace minerals are available in NHANES. All diets included TBHQ antioxidant at 0.014 g/kg.

[1] CocoaVia™ Cardio Health Powder nutirition information available online. For the amount of powder added to CP diets in this study, there was an added 16 g of total carbohydrate, 12 g of dietary fiber, <4 g of protein, 4600 mg Cocoapro™ cocoa bean extract, and 2000 mg cocoa flavanols (including 320 mg of (-)-epicatechin) to the basal diets.

[2] Reference 38: Hintze, K.J.; Benninghoff, A.D.; Ward, R.E. Formulation of the total Western diet (TWD) as a basal diet for rodent cancer studies. *J Ag Food chem* **2012**, *60*, 6736-6742, doi:10.1021/jf204509a.

Table S2. Effects of diet and supplement on Firmicutes:Bacteroidota ratio within each sample site and time point.

Time point	Site	Source	*p*-value
Pre-DSS	Cecal	Diet	0.3404
Pre-DSS	Cecal	Supplement	0.6386
Pre-DSS	Cecal	Diet*Supplement	0.4040
Pre-DSS	Fecal	Diet	**0.0485**
Pre-DSS	Fecal	Supplement	0.3386
Pre-DSS	Fecal	Diet*Supplement	0.1592
Pre-DSS	Mucosal	Diet	0.7882
Pre-DSS	Mucosal	Supplement	0.2539
Pre-DSS	Mucosal	Diet*Supplement	0.1597
Colitis	Cecal	Diet	0.6295
Colitis	Cecal	Supplement	0.9615
Colitis	Cecal	Diet*Supplement	0.1215
Colitis	Fecal	Diet	0.2401
Colitis	Fecal	Supplement	0.4645
Colitis	Fecal	Diet*Supplement	0.8968
Colitis	Mucosal	Diet	0.2663
Colitis	Mucosal	Supplement	0.4121
Colitis	Mucosal	Diet*Supplement	0.6779
Recovery	Cecal	Diet	0.3115
Recovery	Cecal	Supplement	0.1505
Recovery	Cecal	Diet*Supplement	0.8875
Recovery	Fecal	Diet	**0.0061**
Recovery	Fecal	Supplement	0.9548
Recovery	Fecal	Diet*Supplement	0.8201
Recovery	Mucosal	Diet	0.9976
Recovery	Mucosal	Supplement	0.3392
Recovery	Mucosal	Diet*Supplement	0.8084

Data shown are *p*-values for diet and supplement experimental factors and their interaction as determined by a generalized linear model. $p < 0.05$ was considered statistically significant.

Table S3. Pairwise comparisons of diet × supplement on Firmicutes:Bacteroidota ratio for each time point and sample site.

Time point	Site	Pairwise comparison	*p*-value
Pre-DSS	Cecal	AIN/CON vs. AIN/CP	0.7773
Pre-DSS	Cecal	AIN/CON vs. TWD/CON	0.9998
Pre-DSS	Cecal	AIN/CP vs. TWD/CON	0.7479
Pre-DSS	Cecal	AIN/CON vs. TWD/CP	0.9841
Pre-DSS	Cecal	AIN/CP vs. TWD/CP	0.5657
Pre-DSS	Cecal	TWD/CON vs. TWD/CP	0.9938
Pre-DSS	Fecal	AIN/CON vs. AIN/CP	0.9868
Pre-DSS	Fecal	AIN/CON vs. TWD/CON	0.0849
Pre-DSS	Fecal	AIN/CP vs. TWD/CON	0.1631
Pre-DSS	Fecal	TWD/CON vs. TWD/CP	0.3370
Pre-DSS	Fecal	AIN/CON vs. TWD/CP	0.8670
Pre-DSS	Fecal	AIN/CP vs. TWD/CP	0.9717
Pre-DSS	Mucosal	AIN/CON vs. TWD/CP	0.7418
Pre-DSS	Mucosal	AIN/CON vs. AIN/CP	0.9972
Pre-DSS	Mucosal	AIN/CP vs. TWD/CP	0.6239
Pre-DSS	Mucosal	AIN/CON vs. TWD/CON	0.8400
Pre-DSS	Mucosal	AIN/CP vs. TWD/CON	0.9209
Pre-DSS	Mucosal	TWD/CON vs. TWD/CP	0.2743
Colitis	Cecal	AIN/CON vs. AIN/CP	0.6365
Colitis	Cecal	AIN/CP vs. TWD/CP	0.8513
Colitis	Cecal	AIN/CON vs. TWD/CON	0.4825
Colitis	Cecal	AIN/CP vs. TWD/CON	0.9908
Colitis	Cecal	TWD/CON vs. TWD/CP	0.7069
Colitis	Cecal	AIN/CON vs. TWD/CP	0.9776
Colitis	Fecal	AIN/CON vs. AIN/CP	0.9289
Colitis	Fecal	AIN/CON vs. TWD/CON	0.7908
Colitis	Fecal	AIN/CON vs. TWD/CP	0.5408
Colitis	Fecal	AIN/CP vs. TWD/CON	0.9869
Colitis	Fecal	AIN/CP vs. TWD/CP	0.8646
Colitis	Fecal	TWD/CON vs. TWD/CP	0.9705
Colitis	Mucosal	AIN/CON vs. AIN/CP	0.8015
Colitis	Mucosal	AIN/CON vs. TWD/CON	0.7055
Colitis	Mucosal	AIN/CON vs. TWD/CP	0.4672
Colitis	Mucosal	AIN/CP vs. TWD/CON	0.9969
Colitis	Mucosal	AIN/CP vs. TWD/CP	0.9540
Colitis	Mucosal	TWD/CON vs. TWD/CP	0.9916
Recovery	Cecal	AIN/CON vs. AIN/CP	0.7789

Table S3. Pairwise comparisons of diet × supplement on Firmicutes:Bacteroidota ratio for each time point and sample site.

**Time point**	**Site**	**Pairwise comparison**	***p*-value**
Recovery	Cecal	AIN/CP vs. TWD/CON	0.9885
Recovery	Cecal	AIN/CON vs. TWD/CON	0.9343
Recovery	Cecal	AIN/CON vs. TWD/CP	0.3064
Recovery	Cecal	AIN/CP vs. TWD/CP	0.8055
Recovery	Cecal	TWD/CON vs. TWD/CP	0.6622
Recovery	Fecal	AIN/CON vs. AIN/CP	0.9993
Recovery	Fecal	AIN/CON vs. TWD/CON	0.1695
Recovery	Fecal	AIN/CP vs. TWD/CON	0.1618
Recovery	Fecal	TWD/CON vs. TWD/CP	0.9970
Recovery	Fecal	AIN/CON vs. TWD/CP	0.1869
Recovery	Fecal	AIN/CP vs. TWD/CP	0.1757
Recovery	Mucosal	AIN/CON vs. TWD/CON	0.9983
Recovery	Mucosal	AIN/CON vs. AIN/CP	0.9561
Recovery	Mucosal	AIN/CP vs. TWD/CON	0.9028
Recovery	Mucosal	AIN/CON vs. TWD/CP	0.8966
Recovery	Mucosal	AIN/CP vs. TWD/CP	0.9978
Recovery	Mucosal	TWD/CON vs. TWD/CP	0.8168

Data shown are *p*-values for pairwise comparisons for each experimental diet/supplement as determined by a generalized linear model with Tukey HSD post-hoc test for multiple comparisons. FDR $p < 0.05$ was considered statistically significant.

Table S4. Main effects of time point, site, diet and supplement on microbiome alpha diversity.

Factor	Observed	Chao1	Shannon
Site	**<0.0001**	**<0.0001**	**<0.0001**
TP	**<0.0001**	**<0.0001**	**<0.0001**
Site*TP	**0.0169**	**0.0203**	0.2038
Diet	0.1778	0.2047	**0.0129**
Site*Diet	0.8508	0.9063	0.6089
TP*Diet	0.7376	0.6154	0.9155
Site*TP*Diet	0.9229	0.9442	0.1085
Supp	**0.0006**	**0.0005**	0.0829
Site*Supp	0.1338	0.1083	0.7767
TP*Supp	0.6396	0.6578	0.4774
Site*TP*Supp	0.3855	0.3318	0.9358
Diet*Supp	**0.0319**	0.0800	**0.0096**
Site*Diet*Supp	0.8006	0.7990	0.8773
TP*Diet*Supp	0.8819	0.8453	0.6792
Site*TP*Diet*Supp	0.3679	0.4301	0.2457

Data shown are p-values for each experimental factor and their interactions as determined by a generalized linear model. $p < 0.05$ was considered statistically significant.

Table S5. Effects of diet and supplement on alpha diversity within each sample site and time point.

**Site**	**Time point**	**Diet x Supp.**	**Observed**	**Chao1**	**Shannon**
Cecal	Pre-DSS	Diet	0.2494	0.4008	**0.0255**
Cecal	Pre-DSS	Supplement	**0.0012**	**0.0012**	0.8197
Cecal	Pre-DSS	Diet*Supplement	0.2477	0.6084	0.2606
Cecal	Colitis	Diet	0.6299	0.7071	0.4875
Cecal	Colitis	Supplement	0.1588	0.2386	0.1866
Cecal	Colitis	Diet*Supplement	0.5143	0.4797	0.4008
Cecal	Recovery	Diet	0.3556	0.3312	0.3452
Cecal	Recovery	Supplement	0.3800	0.3163	0.7128
Cecal	Recovery	Diet*Supplement	0.0752	0.0724	0.1785
Fecal	Pre-DSS	Diet	0.7482	0.9004	0.1803
Fecal	Pre-DSS	Supplement	0.4209	0.3878	0.4828
Fecal	Pre-DSS	Diet*Supplement	0.5472	0.4230	0.8544
Fecal	Colitis	Diet	0.7091	0.6863	0.7493
Fecal	Colitis	Supplement	**0.0187**	**0.0188**	0.4364
Fecal	Colitis	Diet*Supplement	0.0868	0.2104	0.1341
Fecal	Recovery	Diet	0.5046	0.4968	0.7117
Fecal	Recovery	Supplement	**0.0387**	**0.0370**	0.3018
Fecal	Recovery	Diet*Supplement	0.3329	0.4719	0.3264
Mucosal	Pre-DSS	Diet	0.5897	0.5823	0.5885
Mucosal	Pre-DSS	Supplement	0.5684	0.5225	0.6976
Mucosal	Pre-DSS	Diet*Supplement	0.2306	0.2673	0.0534
Mucosal	Colitis	Diet	0.5446	0.5415	0.3215
Mucosal	Colitis	Supplement	0.3961	0.3749	0.4061
Mucosal	Colitis	Diet*Supplement	0.7838	0.8122	0.2849
Mucosal	Recovery	Diet	0.5874	0.5211	0.0761
Mucosal	Recovery	Supplement	0.7483	0.7809	0.3600
Mucosal	Recovery	Diet*Supplement	0.8847	0.9294	0.6349

Data shown are p-values for diet and supplement experimental factors and their interaction as determined by a generalized linear model. $p < 0.05$ was considered statistically significant.

Table S6. Pairwise comparisons of diet × supplement on alpha diversity for each time point and sample site.

Time point	Site	Diet × Supplement	Chao1	Shannon	Observed
Pre-DSS	Cecal	AIN/CON vs AIN/CP	**0.0289**	**0.0092**	0.7548
Pre-DSS	Cecal	AIN/CON vs. TWD/CON	0.9954	1.0000	0.8324
Pre-DSS	Cecal	AIN/CON vs. TWD/CP	0.2155	0.3126	0.4307
Pre-DSS	Cecal	AIN/CP vs TWD/CON	**0.0213**	**0.0124**	0.2933
Pre-DSS	Cecal	AIN/CP vs. TWD/CP	0.7577	0.3446	0.0778
Pre-DSS	Cecal	TWD/CON vs TWD/CP	0.1607	0.3433	0.9185
Pre-DSS	Fecal	AIN/CON vs AIN/CP	1.0000	0.9988	0.9202
Pre-DSS	Fecal	AIN/CON vs. TWD/CON	0.9096	0.9111	0.6909
Pre-DSS	Fecal	AIN/CON vs. TWD/CP	0.9508	0.9852	0.4657
Pre-DSS	Fecal	AIN/CP vs TWD/CON	0.8924	0.8507	0.9653
Pre-DSS	Fecal	AIN/CP vs. TWD/CP	0.9617	0.9970	0.8347
Pre-DSS	Fecal	TWD/CON vs TWD/CP	0.6349	0.7458	0.9821
Pre-DSS	Mucosal	AIN/CON vs AIN/CP	0.9859	0.9674	0.3407
Pre-DSS	Mucosal	AIN/CON vs. TWD/CON	0.6357	0.5999	0.2885
Pre-DSS	Mucosal	AIN/CON vs. TWD/CP	0.9999	1.0000	0.9095
Pre-DSS	Mucosal	AIN/CP vs TWD/CON	0.8294	0.8566	0.9995
Pre-DSS	Mucosal	AIN/CP vs. TWD/CP	0.9768	0.9627	0.7282
Pre-DSS	Mucosal	TWD/CON vs TWD/CP	0.5962	0.5861	0.6632
Colitis	Cecal	AIN/CON vs AIN/CP	0.5164	0.4367	0.3973
Colitis	Cecal	AIN/CON vs. TWD/CON	0.9954	0.9994	0.9996
Colitis	Cecal	AIN/CON vs. TWD/CP	0.9251	0.8875	0.9609
Colitis	Cecal	AIN/CP vs TWD/CON	0.7122	0.5687	0.5191
Colitis	Cecal	AIN/CP vs. TWD/CP	0.8568	0.8394	0.6767
Colitis	Cecal	TWD/CON vs TWD/CP	0.9862	0.9463	0.9851
Colitis	Fecal	AIN/CON vs AIN/CP	0.0643	**0.0306**	0.3844
Colitis	Fecal	AIN/CON vs. TWD/CON	0.9279	0.7629	0.8288
Colitis	Fecal	AIN/CON vs. TWD/CP	0.4712	0.4581	0.9878
Colitis	Fecal	AIN/CP vs TWD/CON	0.1767	0.1834	0.8513
Colitis	Fecal	AIN/CP vs. TWD/CP	0.6178	0.4181	0.5394
Colitis	Fecal	TWD/CON vs TWD/CP	0.8076	0.9496	0.9459
Colitis	Mucosal	AIN/CON vs AIN/CP	0.9630	0.9739	0.5108
Colitis	Mucosal	AIN/CON vs. TWD/CON	0.9326	0.9256	0.9999
Colitis	Mucosal	AIN/CON vs. TWD/CP	0.9965	0.9976	0.9993
Colitis	Mucosal	AIN/CP vs TWD/CON	0.7374	0.7545	0.6033
Colitis	Mucosal	AIN/CP vs. TWD/CP	0.9926	0.9948	0.4407
Colitis	Mucosal	TWD/CON vs TWD/CP	0.8574	0.8583	0.9982
Recovery	Cecal	AIN/CON vs AIN/CP	0.1975	0.2356	0.6062
Recovery	Cecal	AIN/CON vs. TWD/CON	0.9351	0.9288	0.9921

Table S6. Pairwise comparisons of diet × supplement on alpha diversity for each time point and sample site.

Time point	Site	Diet × Supplement	Chao1	Shannon	Observed
Recovery	Cecal	AIN/CON vs. TWD/CP	1.0000	1.0000	0.9750
Recovery	Cecal	AIN/CP vs TWD/CON	0.4967	0.5727	0.7816
Recovery	Cecal	AIN/CP vs. TWD/CP	0.1540	0.1693	0.3006
Recovery	Cecal	TWD/CON vs TWD/CP	0.9295	0.9026	0.8895
Recovery	Fecal	AIN/CON vs AIN/CP	0.1886	0.1397	0.4793
Recovery	Fecal	AIN/CON vs. TWD/CON	1.0000	0.9968	0.7997
Recovery	Fecal	AIN/CON vs. TWD/CP	0.7083	0.7119	0.7448
Recovery	Fecal	AIN/CP vs TWD/CON	0.1983	0.2067	0.9624
Recovery	Fecal	AIN/CP vs. TWD/CP	0.7016	0.5888	0.9629
Recovery	Fecal	TWD/CON vs TWD/CP	0.7251	0.8310	1.0000
Recovery	Mucosal	AIN/CON vs AIN/CP	0.9938	0.9874	0.9888
Recovery	Mucosal	AIN/CON vs. TWD/CON	0.9812	0.9928	0.4142
Recovery	Mucosal	AIN/CON vs. TWD/CP	0.9932	0.9984	0.9098
Recovery	Mucosal	AIN/CP vs TWD/CON	0.9144	0.9279	0.2376
Recovery	Mucosal	AIN/CP vs. TWD/CP	0.9442	0.9529	0.7333
Recovery	Mucosal	TWD/CON vs TWD/CP	0.9990	0.9992	0.7419

Data shown are *p*-values for pairwise comparisons for each experimental diet/supplement as determined by a generalized linear model with Tukey HSD post-hoc test for multiple comparisons. FDR $p < 0.05$ was considered statistically significant.

Table S7. Effects of diet and supplement at each sample site and time point on unweighted and weighted UniFrac distance beta diversity.

	Pre-DSS				Colitis				Recovery			
**Comparison**	**Unweighted**		**Weighted**		**Unweighted**		**Weighted**		**Unweighted**		**Weighted**	
	R^2	*p*-val	R^2	*p*-val	R^2	*p*-val	R^2	*p*-val	R^2	*p*-val	R^2	*p*-val
Cecal												
Diet × supplement	**0.22**	**0.001**	**0.36**	**0.001**	**0.17**	**0.044**	0.13	0.275	**0.21**	**0.005**	**0.20**	**0.017**
TWD vs. AIN	0.06	0.060	**0.30**	**0.001**	0.06	0.076	0.03	0.582	0.07	0.064	**0.11**	**0.014**
CP vs CON	**0.12**	**0.002**	0.04	0.298	0.07	0.053	0.04	0.368	**0.11**	**0.005**	0.03	0.550
Fecal												
Diet × supplement	**0.21**	**0.001**	**0.30**	**0.001**	0.15	0.150	**0.19**	**0.040**	**0.23**	**0.010**	**0.23**	**0.006**
TWD vs. AIN	0.05	0.165	**0.24**	**0.001**	0.03	0.709	0.09	0.027	0.05	0.223	**0.11**	**0.016**
CP vs CON	**0.11**	**0.001**	0.04	0.234	0.08	0.031	0.04	0.403	**0.11**	**0.010**	0.04	0.327
Mucosal												
Diet × supplement	0.09	0.518	0.12	0.166	0.15	0.086	0.14	0.143	0.09	0.787	0.08	0.856
TWD vs. AIN	0.02	0.836	0.06	0.092	0.04	0.431	0.06	0.154	0.04	0.347	0.03	0.579
CP vs CON	0.04	0.348	0.03	0.515	0.09	0.012	0.04	0.270	0.03	0.752	0.02	0.873

Data shown are the PERMANOVA R^2 and *p*-values for main effects of diet and supplement and their interaction within each site and time point. R^2 and $p < 0.05$ was considered statistically significant.
